# Non-canonical transcriptional start sites in *E. coli* O157:H7 EDL933 are regulated and appear in surprisingly high numbers

**DOI:** 10.1186/s12866-023-02988-6

**Published:** 2023-08-31

**Authors:** Barbara Zehentner, Siegfried Scherer, Klaus Neuhaus

**Affiliations:** 1 Chair for Microbial Ecology, TUM School of Life Sciences, Department of Molecular Life Sciences, Technical University of Munich, Freising, Germany; 2 ZIEL – Institute for Food & Health, Technical University of Munich, Freising, Germany; 3 Core Facility Microbiome, ZIEL – Institute for Food & Health, Technical University of Munich, Freising, Germany

**Keywords:** Transcriptional start sites, EHEC O157:H7 EDL933, Non-canonical TSS, Differential TSS expression, Pervasive transcription

## Abstract

Analysis of genome wide transcription start sites (TSSs) revealed an unexpected complexity since not only canonical TSS of annotated genes are recognized by RNA polymerase. Non-canonical TSS were detected antisense to, or within, annotated genes as well new intergenic (orphan) TSS, not associated with known genes. Previously, it was hypothesized that many such signals represent noise or pervasive transcription, not associated with a biological function. Here, a modified Cappable-seq protocol allows determining the primary transcriptome of the enterohemorrhagic *E. coli* O157:H7 EDL933 (EHEC). We used four different growth media, both in exponential and stationary growth phase, replicated each thrice. This yielded 19,975 EHEC canonical and non-canonical TSS, which reproducibly occurring in three biological replicates. This questions the hypothesis of experimental noise or pervasive transcription. Accordingly, conserved promoter motifs were found upstream indicating proper TSSs. More than 50% of 5,567 canonical and between 32% and 47% of 10,355 non-canonical TSS were differentially expressed in different media and growth phases, providing evidence for a potential biological function also of non-canonical TSS. Thus, reproducible and environmentally regulated expression suggests that a substantial number of the non-canonical TSSs may be of unknown function rather than being the result of noise or pervasive transcription.

**Supplementary Information:**

The online version contains supplementary material available at 10.1186/s12866-023-02988-6.

## Introduction

The primary transcriptome comprises the entirety of canonical mRNA molecules present in an organism. Analyses targeting the bacterial primary transcriptomes included high-throughput identification of transcription start sites (TSS) during the last years. Such experiments revealed an unexpected complexity of the bacterial transcriptional landscapes containing a large number of non-canonical transcripts. This, in turn, revealed massive antisense (as), intra- and intergenic TSS [e.g., 1, 2–4]. The functionality of the unusual transcription start sites was analyzed in some instances. Thereby, asTSS were identified to promote expression of overlapping protein coding genes [5, 6] or of functional asRNA [7]. Additionally, intergenic signals gain in importance as the significance of small intergenic genes [8–10] and intergenic small RNAs [11] is increasingly recognized. Lastly, intragenic TSS might be reasonable signals for the expression of protein isoforms [12] and out-of-frame alternative products [13, 14].

Despite some well characterized examples and although the precision of high-throughput methods using next-generation sequencing technologies is found to be high, there have been doubts about the significance of the unexpected transcriptional signals [15]. Pervasive transcription, describing random transcriptional activity of the polymerase throughout the bacterial genome, is commonly used as argument to generally reject functionality of non-canonical transcripts [16]; however, a regulatory function of pervasive transcripts has recognized by others [17]. Nevertheless, missing inter-strain specific reproducibility is also used to dismiss the importance of such signals [18]. Interestingly, 10–20% of the genes in every taxonomic group are taxonomically restricted, lacking homologs in other species, and seem to be important for species specific adaptation processes [19]. Perhaps, that is also true for taxonomically restricted TSSs, which should therefore not generally be dismissed as being pervasive, since missing homology does not equal missing function.

Differential RNA sequencing [dRNA-seq, 1], a method widely used since its publication [e.g., 20, 21, 22], revolutionized the analysis of the primary bacterial transcriptome. More recently, L Ettwiller, J Buswell, E Yigit and I Schildkraut [23] published an alternative approach termed Cappable-seq, allowing to determine TSS genome wide at single base resolution after specifically enriching for the 5’ end of primary transcripts. By using a triphosphate specific capping enzyme, Cappable-seq enables a highly efficient tagging of primary 5’ triphosphorylated mRNA transcripts with a biotin cap, followed by direct enrichment with streptavidin beads and subsequent next generation sequencing (Supplementary Figure S1). This approach was used to identify transcription start sites of several bacterial species including the model organism *E. coli* [23], *Streptococcus pneumoniae* [24], and the endosymbiont *Wolbachia* [25]. However, most of these studies did not analyze differential expression and regulation of gene expression based on differential TSS signals. Here we examine two growth media, low pH, high salt in two growth conditions in order to learn about the TSS regulation in a pathogenic *E. coli* strain.

The human enterohemorrhagic pathogen *Escherichia coli* O157:H7 (further designated EHEC) was first identified in 1983 as the causal agent in undercooked hamburger meat. The pathogen causes symptoms like watery diarrhea and a severe enterohemorrhagic colitis, which can end up in an acute renal failure associated with the hemolytic uremic syndrome [HUS; 26, 27, 28]. The main pathogenicity factors of EHEC are Shiga toxins [Stx1, Stx2; 29] and a type III secretion system (T3SS) encoded on the locus of enterocyte effacement pathogenicity island [LEE; 30]. Due to its importance as pathogenic bacterium, EHEC is well examined. Although different studies addressed the transcriptome [31], translatome [32, 33], and proteome [34, 35] of EHEC, a high-throughput analysis of the primary transcriptome of EHEC is missing. Furthermore, previous work from our group suggested that EHECs strains contain a number of additional genes, which have not been annotated using standard methods and are not found in *E. coli* K12. This comprises genes found using transcriptome profiling [31, 36], but also small genes [37, 38], genes which supposedly were non-coding RNA, but seemed to code for a protein nevertheless [39] and overlapping genes (i.e., the coding region of an open reading frame is embedded in a coding region of a different open reading frame [6, 40–44]). All of these genetic elements need transcriptional start sites for expression and regulation. Therefore, we conducted Cappable-seq on total RNA for *E. coli* O157:H7 strain EDL933 to analyze canonical transcription start sites (TSS) for annotated genes (gTSS) as well as non-canonical TSS, such as TSS antisense to (asTSS) and sense within annotated genes (internal, iTSS), as well as new TSS in intergenic regions without relation to annotated genes (orphan TSS, oTSS). Additionally, TSS were examined in non-stress and stress conditions to analyze comparatively differential expression patterns of transcribed regions in bacterial genomes, potentially indicating regulation and, therefore, suggesting biological function. Such a thorough analysis should give further insights in the transcriptional landscape of this bacterium and will allow drawing conclusions that are more general. Previous experiments have shown that low pH and high salt led to the expression of many novel genetic elements [45]. Thus, low pH and high salt, in combination with a minimal and a complex medium, where considered as most interesting. However, TSS signals are somewhat prone to noise and, thus, some findings of Cappable seq are questioned due to this. The high number of signals found is astonishing. Therefore, we used extensive datasets of four different conditions (two media, low pH, high salt), both in exponential and stationary growth, with biological triplicates and one technical replicate in order to ensure that the observed signals are proper signals. To our knowledge, such an extensive analysis has not been conducted elsewhere.

## Materials and methods

### Bacterial strains and cultivation conditions

*Escherichia coli* O157:H7 EDL933 was used throughout this study. This strain is from the original EHEC outbreak and had been obtained from Collection de l’Institute Pasteur [CIP 106327 = WS 4202; 46, 47]. Cells were cultivated in LB Medium (10 g/L tryptone, 5 g/L yeast extract, 5 g/L NaCl) or M9 minimal medium (6 g/L Na_2_HPO_4_ (anhydrous), 3 g/L KH_2_PO_4_, 1 g/L NH_4_Cl, 0.5 g/L NaCl, 3 mg/L CaCl_2_, 1 mL/L 1 M MgSO_4_ (sterile), 8 mL/L 25% glucose (sterile), 5 mL/L 20% casamino acid (sterile), 0.1 mL/L 0.5% thiamine (sterile)). LB was supplemented with 4 mM L-malic acid or 500 mM sodium chloride for acid and salt stress, respectively. Liquid cultures (50 mL medium in 500-mL baffled flasks) were inoculated with an overnight culture of optical density at 600 nm (OD_600_) equal to 0.03 (LB based media) or with a constant volume of 500 µL (M9 medium). Cells were cultivated at 37 °C with shaking at 150 rpm.

### Isolation of total RNA

Cells were harvested at defined OD_600_ values by centrifugation (9000×*g*, 5 min, 4 °C, Supplementary Table S1) and cell pellets were frozen in liquid nitrogen and stored at − 80 °C. RNA was isolated using Trizol (Invitrogen, Thermo Fisher). All steps were conducted on ice unless otherwise stated. Cell pellets were resuspended in Trizol (see Supplementary Table S1 for amounts used) and mechanically disrupted by bead-beating (0.1 mm Zirconia-beads, FastPrep-24, three times for 6.5 m/s for 45 s with 5 min rest on ice between the runs). Upon disruption, the cell lysates were incubated for 5 min at room temperature (RT). Afterwards, 0.2 Vol of cooled chloroform per initial amount of Trizol was added, samples were mixed vigorously (15 s by vortexing) and incubated for 5 min at RT. Phases were separated by centrifugation (12,000×*g*, 15 min, 4 °C). The upper phase was recovered and nucleic acids were precipitated with 0.1 Vol of the upper phase of 3 M NaOAc (Invitrogen), 1 µL glycogen (RNA grade, Thermo Fisher) and 1 Vol of 2-propanol (RT, Carl Roth) for 1 h at − 20 °C. RNA was pelleted (12,000×*g*, 10 min, 4 °C) and washed twice with 1 mL 80% ethanol (12,000×*g*, 5 min, 4 °C). The remaining alcohol was collected (20–30 s centrifugation) and removed. The RNA pellet was dried (RT, max. 15 min) and subsequently dissolved in 40 µL RNase free H_2_O (Millipore).

### DNase digest

DNA in RNA samples was digested with Ambion Turbo DNase (Invitrogen, Thermo Fisher) according to the manufacturer’s instructions. DNase was inactivated with 15 mM EDTA (final concentration) at 75°C for 10 min. The RNA was recovered by ethanol precipitation. Absence of DNA was verified with a standard Taq-PCR (NEB) using primers rrsh-F (5’ AATGTTGGGTTAAGTCCCGC 3’) and rrsh-R (5’ GGAGGTGATCCAACCGCAGG 3’) amplifying a segment of the 16 S rDNA gene using the following PCR temperature program: initial denaturation 95 °C for 2 min, 30 cycles with 95 °C for 30 s, 60 °C for 30 s and 68 °C for 28 s, final elongation 68 °C for 5 min. The quality of the RNA was checked with capillary gel electrophoresis (Bioanalyzer 2100, RNA 6000 Nano Kit) and the concentration was measured with a Nanodrop 1000.

### Determination of transcriptional start sites using Cappable-seq

Total RNA (min. 10 µg, DNA depleted) was applied to the Cappable-seq sample preparation procedure [23] adapted with a tag-RNA-seq approach [48] (conducted by Vertis Biotechnologie AG, Freising, Supplementary Figure S1). Briefly, 5′ triphosphorylated RNA were reversible capped with DTB-GTP (3′ desthiobiotin-TEG-guanosine 5′ triphosphate) by the vaccinia capping enzyme. All transcripts are fragmented and size selected (> 70 nt). 5′ capped RNA fragments are captured with streptavidin beads and separated from uncapped RNA. 3′ ends are poly(A) tailed with a poly(A) polymerase and 5′ monophosphorylated contaminants are ligated to 5′ Illumina TruSeq sequencing adapters, which carry a unique sequence tag 1 (PSS-set). The biotin cap is enzymatically removed with Cap-Clip Acid Pyrophosphatase (de-capping) and newly exposed 5′ monophosphates of previous primary transcripts are ligated to 5′ Illumina TruSeq sequencing adapters carrying the sequence tag 2 (TSS-set). Oligo(dT)-adapter primer are used for synthesis of first-strand cDNA with M-MLV reverse transcriptase. The cDNA is PCR-amplified with primers binding at the 3′ end of the first-strand cDNA exhibiting a biotinylation. The amplification products are enzymatically fragmented and size selected using streptavidin beads (size range: 100–300 bp). Illumina sequencing adapters (3′) are ligated and the cDNA is finally amplified in a PCR reaction. PCR libraries are pooled, size fractionated (200–500 bp), and sequenced on an Illumina NextSeq 500 system (single end, 75 bp).

### Evaluation of sequencing data

Reads were demultiplexed with cutadapt [49] and PSS-/TSS-set separated raw sequencing reads were quality trimmed with the program Trimmomatic [50] by removing low quality reads as well as reads with Poly-A-80-, Poly-T-80, Poly-G-80- and Poly-AG-tail. The remaining reads were mapped to the genome of *Escherichia coli* O157:H7 EDL933 (NCBI accession no. NZ_CP008957) using bowtie2 [51]. Tool version numbers, input file instructions and settings for Trimmomatic, and bowtie2 are given in Supplementary File S1.

### Bioinformatic TSS determination

Two programs, provided by L Ettwiller, J Buswell, E Yigit and I Schildkraut [23], were used to determine transcriptional start sites. Briefly, the program bam2firstbasegtf.pl trims mapped sequencing reads to the most 5’ base leaving a 1 bp long “read” and calculates its relative read score RRS (${RRS}_{io}=\frac{{n}_{io}}{N}*{10}^{6}$ with n_io_ the number of reads at position i and orientation o and N the total number of all mapped reads in the respective condition). Positions with an RRS of at least 1.5 (minRRS = 1.5) are maintained. The program cluster_tss.pl clusters putative TSS positions from the first program dynamically within a 5 bp distance and the position with the highest RRS remains as TSS. Execution details are shown in Supplementary File S1. A TSS was defined as reliable for any TSS signal present in all three biological replicates of the same analyzed condition.

The 5’ UTRs analysis was conducted for TSS within a maximum distance of 500 nucleotides between TSS and start codon at minRRS = 5. The optimal upstream distance was evaluated and an upstream region of at most 250 bp from the start codon of the respective gene or a downstream region (1/3 of the gene length downstream of the start codon of the respective gene, but not more than 200 bp) was screened for gene associated TSS.

### Comparison of EHEC TSS with ***E. coli*** K12 TSS

Homologous genes between *E. coli* str. K-12 substr. MG1655 (NC_000913.3) and *E. coli* O157:H7 EDL933 were searched with Diamond blastp using the e-value cutoff 10^− 5^. Data for TSS and the associated genes deposited in the RegulonDB [v. 10.5, 52] for *E. coli* str. K-12 substr. MG1655 were collected. TSS data for *E. coli* O157:H7 EDL933 are from this study. The distance between start codon and transcription start site was calculated for genes present in both *E. coli* strains, respectively. The difference between the distances of each individual strain data set was taken to estimate the reliability of TSS determination. A small distance of the TSS found between a homolog present in EHEC and in *E. coli* K12, was taken as indication for a reliable determination. To make an example, the distance between the start codon and the TSS might be 5 bp in EHEC, but 7 bp in *E. coli* K12; thus, the difference of distances equals 2 bp.

### Sequence logo

Upstream regions (100 bp) of gene-associated transcription start sites were analyzed for conserved patterns using WebLogo 3 [53]. A randomly selected number of genome positions and the associated upstream regions were used as negative control for this analysis. Tool version numbers, input file instructions and settings are given in Supplementary File S1.

### Determination of internal TSS and differentiation from background

Putative transcription start sites within annotated genes (between 20 bp downstream of start codon to end of gene) were selected at minRRS = 1.5. To estimate whether these putative TSSs show signals clearly above the background, we searched for the highest background signal within each annotated gene. For this, we firstly excluded all positions which are reproducibly associated with an annotated gene (upstream or downstream, see above) or within the respective gene. The remaining positions were screened for the one signal with the highest relative read score (highest RRS_noise_). For each putative iTSS, a signal-to-noise ratio was calculated using the formula $\frac{S}{N}=\frac{{RRS}_{iTSS}}{{RSS}_{noise}}$ (RRS_iTSS_ is the relative read score for the internal TSS). This procedure was conducted for all three replicates separately. If this signal-to-noise ratio (S/N) exceeded the threshold 1.5 in all replicates, i.e., the signal of the TSS is 1.5 times higher than the background, the TSS was considered a true TSS and not noise, e.g., from degradation.

### Determination of antisense transcription start sites (asTSS)

Putative transcription start sites antisense to annotated genes (including 100 bp upstream and downstream of start and stop codon of the annotated gene) were selected at minRRS = 1.5. In some cases, the asTSS was positioned inside another annotated gene on the same DNA strand, e.g., due to genes following each other in operons. Again, such signals were only considered as ‘true’ TSS if the signal-to-noise ratio according to the previous section was above the threshold.

### Differential regulation of transcription start sites

Absolute read counts of all identified TSS were used to assess differential regulation of transcription start sites with the *Bioconductor* package *edgeR* [v 3.28.0, 54, 55]. The *tagwise dispersion* of the dataset was calculated with the *estimateDisp* function using suitable design matrices for different comparisons created with *model.matrix*. Significant differences of stress conditions (minimal medium, LB + L-malic acid, LB + NaCl) compared to the non-stress condition LB in the respective growth phase, or significant differences of TSS signals between growth phases were determined. p-values were adjusted using Benjamini-Hochberg adjustment method within the *topTags* function of *edgeR*. Significant up- or downregulation was assumed for logFC > |2| (equates a fold change of 4) with a false discovery rate FDR < 0.05.

### RT-qPCR

Reverse transcription of RNA was performed using SuperScript III Reverse Transcriptase (Invitrogen, Thermo Fisher) according to the manufacturer. Briefly, 500 ng total RNA, 10 pmol gene-specific primer and 1 µL dNTP mix (10 mM each dNTP) was heated at 65 °C for 5 min in a reaction volume of 13 µL. After incubation on ice for at least 1 min, 4 µL 5×First-Strand Buffer, 1 µL 0.1 M DTT, 1 µL SUPERase•In RNase Inhibitor (Invitrogen, Thermo Fisher) and 100 U reverse transcriptase was added and first strand cDNA was synthesized at 55 °C for 60 min. Inactivation was carried out at 70 °C for 15 min. Samples were stored at − 20 °C. For each reverse transcription reaction, a no-RT control was processed, where reverse transcriptase was replaced by H_2_O to verify absence of genomic DNA in RNA samples.

Quantitative PCR (qPCR) was performed on a Biorad CFX96 Touch Real-Time PCR Detection System in PCR stripes in a 10-µL reaction containing 5 µL SYBR™ Select Mastermix, 400 nM forward and reverse primer for the respective amplicons (Supplementary Table S2), and 1 µL template. The reaction was performed using the following cycling conditions: 2 min at 50 °C (UDP activation), 2 min at 95 °C (initial denaturation), 40 cycles of denaturation (15 s at 95 °C), annealing (15 s at the optimal annealing temperature, Supplementary Table S2﻿), and elongation (1 min at 72 °C). A subsequent melting curve was recorded (60 to 95 °C) to monitor specificity of the amplicons. Each qPCR run contained a non-template control (H_2_O instead of the template) and a positive control (genomic DNA as template). Primer efficiencies were determined with genomic DNA (technical triplicates). Expression profiling was conducted in biological triplicates with three technical replicates on cDNA from RNA isolated at the indicated conditions. Data was evaluated with the software Bio-Rad CFX Maestro. Relative quantities (ΔCq) for all samples of each gene of interest (GOI) were calculated with the formula ${\varDelta Cq}_{sample\left(GOI\right)}= {E}^{{Cq}_{\left(min\right)}-{Cq}_{\left(sample\right)}}$ with the primer efficiency E (E = % Efficiency * 0.01 + 1), the average Cq value for the sample with the lowest Cq for GOI Cq_(min)_, and the Cq for the sample Cq_(sample)_ (average of technical replicates). The normalized expression ΔΔCq was calculated regarding the relative quantities of the reference gene *cysG* with the formula $\varDelta \varDelta Cq\left(GOI\right)=\frac{{\varDelta Cq}_{sample \left(GOI\right)}}{{\varDelta Cq}_{sample \left(cysG\right)}}.$ Mean values and standard deviations were calculated. Significant different expression between iTSS and gTSS was tested with a one-tailed Welch two sample t-test and different expression of a gTSS/asTSS in different conditions was tested with a one-tailed paired t-test.

### Bioinformatic promoter prediction

The program bTSSfinder [56] was used with standard settings and scoring thresholds for *E. coli* to predict promoter sequences. Input sequences were 251 bp long spanning 200 bp upstream of the TSS and 50 bp downstream of the TSS.

### Promoter activity assay

Putative promoter sequences predicted with bTSSfinder were cloned with standard cloning techniques in the promoterless *gfp*-reporter plasmid pProbe-NT using primers listed in Supplementary Table S3 and the restriction enzymes *Sal*I and *EcoR*I. Cloned promoter sequences span lengths between 51 and 101 bp. The vector constructs were transformed into *E. coli* Top10 where the assay was conducted.

Overnight cultures of *E. coli* Top10, *E. coli* Top10 + pProbe-NT + promoter, and *E. coli* Top10 + pProbe (empty vector) were used to inoculate 10 mL growth medium (LB medium or LB medium + 450 mM NaCl, as indicated for the respective promoter construct) in a 1:100 ratio. Cells were cultivated at 37 °C and 150 rpm and harvested by centrifugation (2 min, 6600×*g*, 4 °C) at an optical density of OD_600_ = 0.5–0.6. The cell pellet was washed once in 1 mL 1×PBS (137 mM NaCl, 2.7 mM KCl, 10 mM Na_2_HPO_4_, 1.8 mM KH_2_PO_4_) and finally resuspended in 1 mL 1×PBS. Cells were diluted 1:10 and OD_600_ was measured. Fluorescence of 200 µL diluted cells was measured in four technical replicates (Wallac Victor^3^, Perkin Elmer, excitation: 485 nm, emission: 535 nm, measuring time 1 s). The mean fluorescence was normalized to OD_600_ = 1 and self-fluorescence of *E. coli* was subtracted. The mean and standard deviation of three biological triplicates was calculated and statistical significances between empty vector constructs and promoter constructs were evaluated with a two tailed Welch two sample t-test (significance level α = 0.05).

## Results

### Reliability of TSS identification using a modified Cappable-seq protocol

**Fig. 1:**
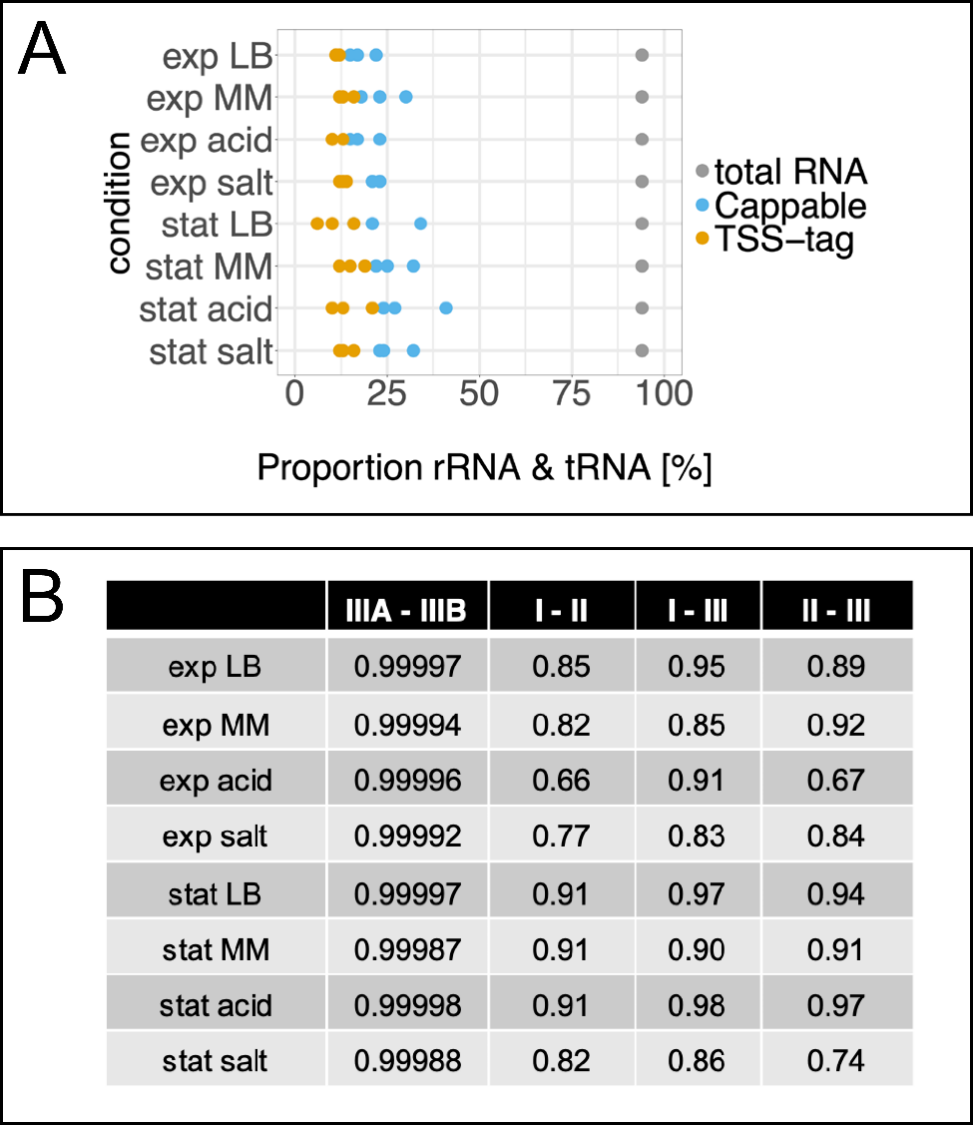
Sequencing details. **A** Proportion of reads mapping to rRNA and tRNA in tag-RNA-seq enriched Cappable-seq RNA for different experimental conditions (as indicated; MM, minimal medium; acid, LB + malic acid; salt, LB + sodium chloride, exp, exponential phase; stat, stationary phase). In Cappable-seq RNA (blue, TSS- and PSS-tag data combined) between 15 to 41% and in TSS-tag enriched Cappable-seq RNA (orange, TSS-tag) about 6 to 21% of the reads match to rRNA and tRNA, respectively. In contrast, total RNA contains about 94% rRNA and tRNA (gray) according to AJ Westermann, SA Gorski and J Vogel [57]. This indicates indirect rRNA and tRNA depletion by combined use of Cappable-seq and TSS-tag-RNA-seq enrichment. **B** Reproducibility of Cappable-seq indicated by the Pearson correlation coefficient *r*. All mapped reads were trimmed to the most 5’ base. For positions with reads, a relative read score was calculated and compared between replicates by calculating the Pearson correlation coefficient *r*. Cappable library III was sequenced twice to obtain technical replicates (IIIA and IIIB), which were merged later to data set III. Data sets I, II and III represent independent biological replicates

We used a modified Cappable-seq of L Ettwiller, J Buswell, E Yigit and I Schildkraut [23], which includes the tag-RNA-seq approach of N Innocenti, M Golumbeanu, AF d’Hérouel, C Lacoux, RA Bonnin, SP Kennedy, F Wessner, P Serror, P Bouloc and F Repoila [48] to determine transcriptional start sites of *E. coli* O157:H7 EDL933 in eight different conditions in biological triplicates. The conditions included LB, minimal medium, LB supplemented with L-malic acid, LB supplemented with sodium chloride and measuring in the exponential and stationary growth phase, respectively (Supplementary Figure S1). Additionally, one Cappable-seq library was sequenced twice to provide a technical replicate (IIIA and IIIB) resulting in overall 32 evaluable datasets. The efficiency of the protocol was verified. Here, disturbing processed transcripts mapping to rRNA and tRNA regions were found to be reduced from theoretically 94% in total RNA [57] to on average 13% in enriched samples (Fig. 1A). Furthermore, highest pairwise Pearson’s product moment correlation coefficients indicate excellent reproducibility of technical (*r* > 0.999) and biological replicates (between *r* = 0.66 and *r* = 0.95 for exponential; between *r* = 0.74 and *r* = 0.98 for stationary phase samples; Fig. 1B).

Transcription start sites were determined with a suite of programs published by L Ettwiller, J Buswell, E Yigit and I Schildkraut [23] using their default selection criteria (according to the methods section). Between 8,161 and 12,307 reproducible TSS (i.e., TSS present in all three biological replicates) were identified in a single condition of the eight analyzed. Combining all eight conditions, a total of 19,975 TSSs are found in *E. coli* O157:H7 EDL933 (Supplementary Table S4). Thereof, 1,140 are associated with regions coding for rRNA or tRNA and were not considered in further analysis.

**Fig. 2:**
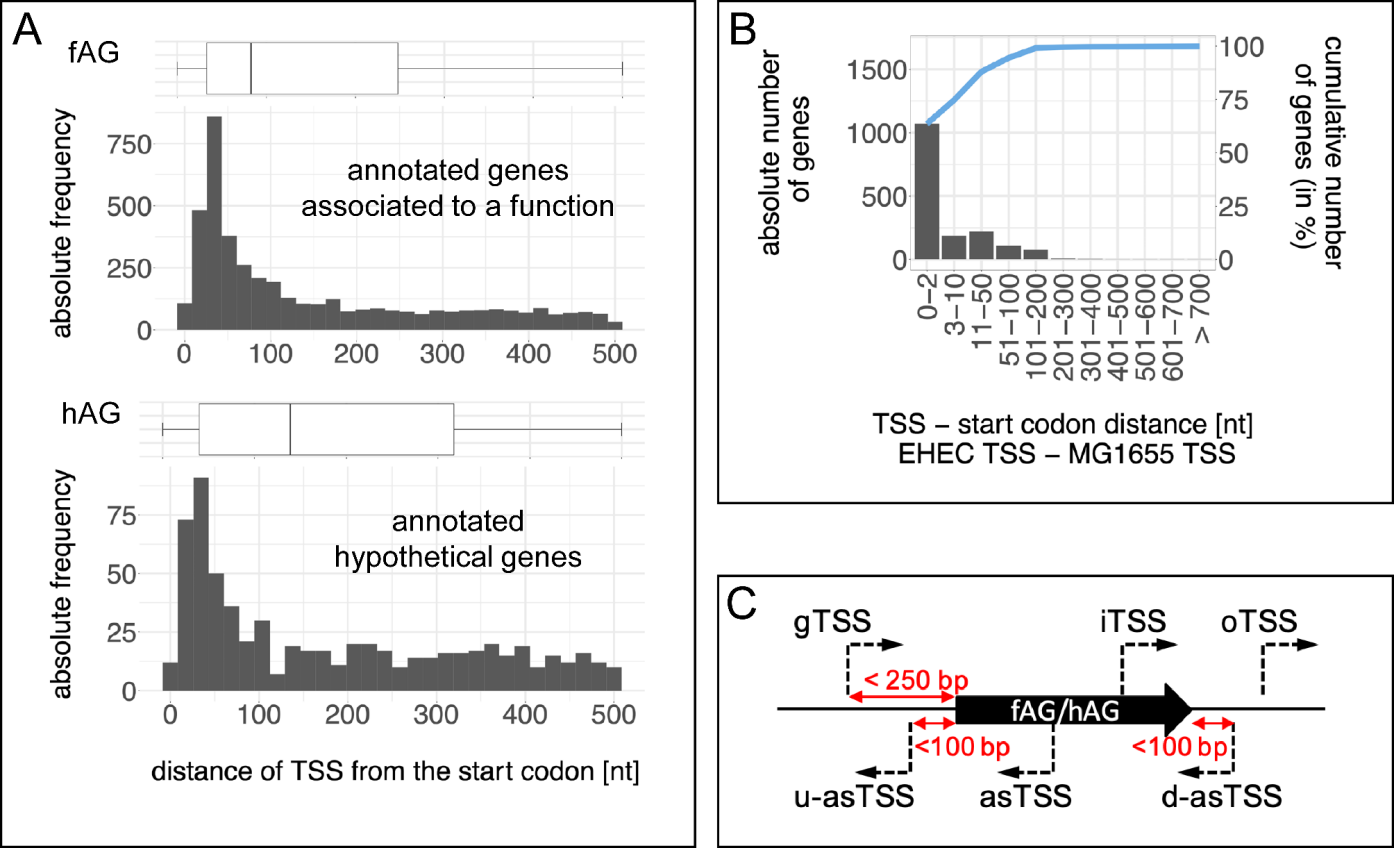
Reliability of TSS identification. **A** Distribution of the distances of the TSS (minRRS ≥ 5) to the start codon within a 500-bp window upstream of 4342 annotated genes associated to a function (fAG, upper panel) and 657 hypothetical genes (hAG, lower panel). The distance between TSS and start codons is given in nucleotides (nt). Box-plots above each panel display minimum (0 bp), maximum (500 bp), 25th percentile (33 and 40 bp), median (83 and 139 bp), and 75th percentile (247 and 317 bp) of the 5′ UTR lengths, respectively. **B** Comparison of TSS between EHEC and *E. coli* K12 MG1655. TSS of 1682 homologous genes of EHEC and *E. coli* K12 MG1655 were analyzed. The frequency of genes with a deviation (specified below each bin) of the distance between start codon and TSS is shown as absolute numbers (left y-axis) and as cumulative function (right y-axis). **C** Classification scheme for TSS used in this study. TSS groups comprise ‘gTSS’, gene associated TSS upstream of the start codon of annotated genes; ‘iTSS’, sense internal TSS within annotated genes; ‘asTSS’, TSS antisense of the coding region of annotated genes or their 5’ UTR (u-asTSS) or 3’ UTR (d-asTSS); and ‘oTSS’, orphan TSS without association to annotated genes

We conducted a stringent 5’-UTR analysis for canonical TSS of annotated genes characterized either as ‘functional’ (fAG) or ‘hypothetical’ (hAG) in the genome of EHEC (GenBank accession NZ_CP008957, annotation of 2017/02 includes 4,525 fAGs and 973 hAGs) to estimate an appropriate distance range for TSS being located upstream of the respective genes’ annotated start codon (Fig. 2A). The most abundant UTR lengths are between 35 and 52 bp, independent on the gene category, which is in concordance with reports of diverse Proteobacteria [1, 58] and supports the reliability of TSS identification. Since 75% of analyzed 5’UTRs for fAGs and hAGs are up to 247 and 317 bp, respectively, a maximum 5’UTR of 250 bp was permitted in further analysis.

We identified for 2,987 annotated genes 5,567 unique TSS. In particular, 4,866 TSS are upstream of fAGs and 763 TSS are upstream of hAGs, with some TSS present in both groups (Supplementary Table S5). This indicates that on average every second gene has more than one TSS. Remaining annotated genes lack a TSS in close proximity (250 bp upstream). However, a wrongly annotated start codon might be the reason for some, since 466 TSSs were identified downstream of the assumed start codons. Further, some genes are arranged in operons and only the first gene might have a TSS and, in addition, some genes are not expressed in the conditions analyzed here.

The reliability of TSS detection was verified further by comparing the distance of the start codon to the TSSs for 1682 homologous genes between EHEC and *E. coli* K12 MG1655 (Fig. 2B, Supplementary Table S5). We found that the distance between start codon and TSS differs by ≤ 2 nucleotides for 64% and by ≤ 10 nucleotides for 75% of the homologous genes analyzed. Thus, a high precision of the method can be inferred. Furthermore, we compared the EHEC TSSs for the genes *qseB* and *lpp* from known data with TSSs identified here. The genome positions 3,996,856 given for the TSS of *qseB* [59] and 2,451,556 for *lpp* [60], respectively, exactly match the TSS determined here, again reinforcing the accuracy of TSS identification in our Cappable-seq experiments.

In summary, Cappable-seq was successfully conducted and transcription start sites in *E. coli* O157:H7 EDL933 were identified genome wide in a highly precise manner. In further analysis, we focused on the reliable identification of the non-canonical transcription start sites, i.e. asTSS, iTSS, and oTSS (Fig. 2C).

### Identification of TSS antisense to, and in intergenic regions of annotated genes

Data visualization of mapped sequencing reads showed that conspicuous signals for TSSs are also located in regions where no TSS would be expected based on current knowledge for genome annotations, namely asTSS and oTSS.

**Fig. 3:**
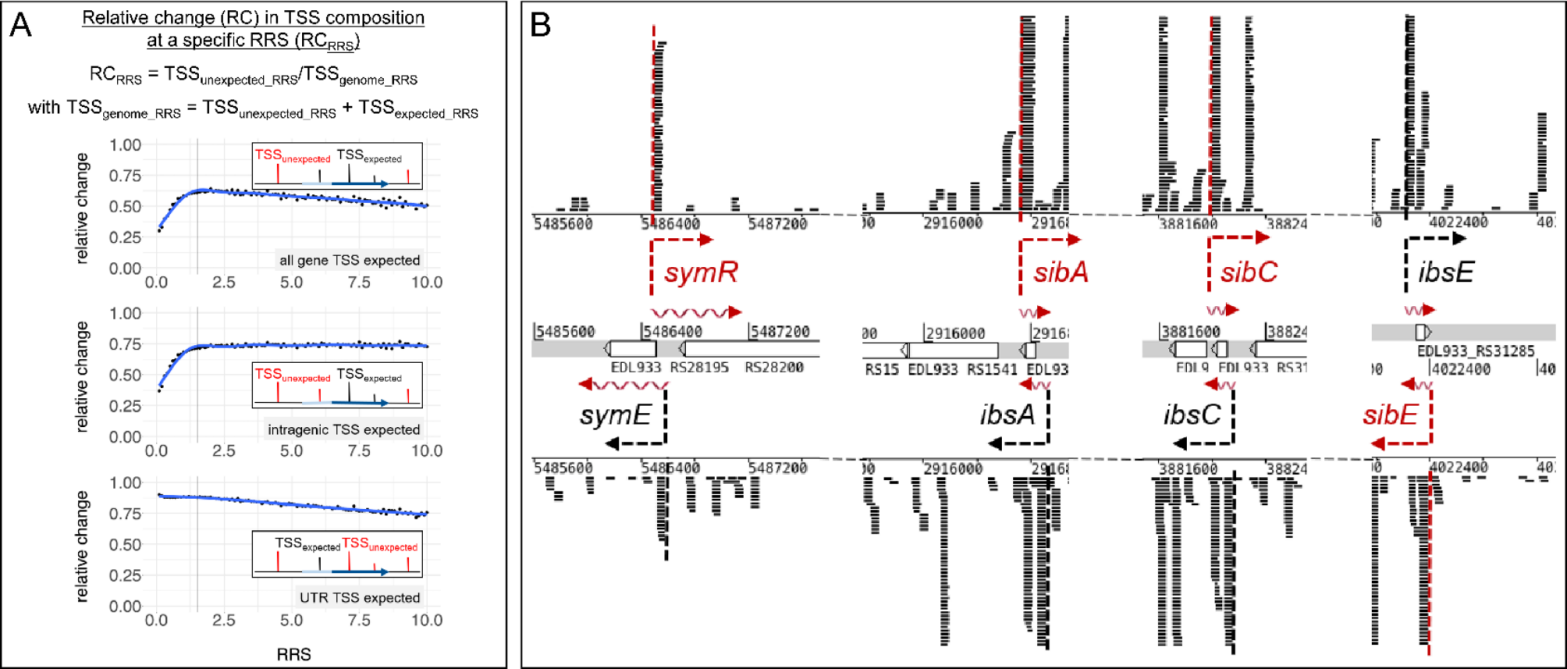
Cutoff re-evaluation (RRS) and antisense TSS. **A** Cutoff re-evaluation. The relative change (RC) of the TSS composition in unexpected genomic positions compared to all genome positions is shown depending on the relative read score (RRS) cutoff (RC_RRS_ = TSS_unexpected_RRS_/TSS_genome_RRS_). TSS_genome_ includes all possible expected and unexpected TSS signals (TSS_genome_ = TSS_expected_ + TSS_unexpected_). The above-mentioned relative change is shown for three possibilities of TSS_expected_: TSS in intragenic region (e.g., degradation signals) and in the 5’ UTR (gTSS, upper panel); TSS only in intragenic regions (middle panel); TSS only in the 5’ UTR (lower panel). A cubic square smooth function (blue line) is placed on the data (dots). A relative read score of 1.5 is indicated with black vertical lines in each panel. The insets exemplary illustrate the analyzed TSS in each case. Black vertical bars, expected TSS in gene regions; red vertical bars, TSS in unusual location, unexpected TSS; dark blue horizontal arrows, coding region of a gene; light blue horizontal bars, 100 bp long 5’ UTR of a gene. **B** Genomic localization and Cappable-seq sequencing reads for the cis-acting regulatory RNAs *symR*, *sibA*, *sibC*, and *sibE*. Transcription start sites are indicated with dashed arrows and lines for the annotated gene (black) and the antisense RNA (red). Reads of replicate I in exponential phase, LB, are shown

First, we wanted to know whether the applied cutoff for TSS determination is suitable to identify reliably any asTSS and oTSS (Fig. 3A). Towards this end, the number of putative TSSs depending on their relative read scores (RRS) were selected throughout the genome (TSS_genome_). Next, TSS were selected only in regions, where we commonly would expect a TSS (TSS_expected_). These regions are defined as 100 bp upstream of an annotated gene, constituting a conservative region for the 5’ UTR, and intragenic regions constituting degradation signals of RNA originating from annotated genes. To obtain the putative TSSs in unexpected regions (TSS_unexpected_), we deleted all TSS_expected_ from the list of TSS_genome_. Next, histograms were built for TSS_genome_ and TSS_unexpected_ in 0.1 RRS-steps and the variation of the number of putative transcription start sites at each step was calculated (i.e., relative change RC at a specific RRS: RC_RRS_ = TSS_unexpected_RRS_/TSS_genome_RRS_). The RC shows at which RRS a high or low fluctuation (corresponding to a low and high RC, respectively) in the TSS composition between the two sets is found. We expect for informative TSSs in unusual positions a high RC indicating low variation. Thus, putative TSSs which are found in both sets (i.e., TSS_unexpected_ and TSS_genome_), do not represent, for example, degradation. Looking at an RRS range from zero to 10, a high relative change is detected at the local maximum of the curve of about 0.61 for cutoff 1.5 (Fig. 3A, **upper panel**). The sharp drop of the RC towards zero can be explained by a high number of putative TSS originating from intragenic regions comprising background noise/degradation (Fig. 3A, **middle panel**), whereas a slighter decrease towards 10 arises from putative TSS positioned in the UTRs, thus representing probably true TSS (Fig. 3A, **lower panel**). Based on this analysis, we assume that a TSS cutoff of 1.5 is appropriate to distinguish between background/degradation and genuine TSS for asTSS and oTSS.

In total, 7,045 asTSSs were reproducibly identified. Due to the high gene density in bacterial genomes, 1,078 asTSS selected as described in the methods section are most likely associated to annotated genes and do not represent asTSS, reducing the number of putative asTSS to 5,967 which are located antisense to 3,366 annotated genes (Supplementary Table S4, Supplementary Table S6). Thereof, 4,685 are directly opposite to annotated genes (more than two thirds), whereas the residual TSSs are located in the annotated genes’ 5’ or 3’ UTRs (Fig. 2C). The reliability of asTSS identification was examined by comparing known TSSs of functionally characterized cis-acting antisense RNAs in *E. coli*. We checked seven cis-regulatory antisense RNAs identified in *E. coli* and found exact TSS matches for all of these [SymR, SibA-E, RdlD, 61, 62, 63, examples shown in Fig. 3B].

In addition to the above, 1,130 TSS with no relation to annotated genes can be considered as oTSS (Supplementary Table S4). These TSS may represent a new source for hitherto neglected expressed transcripts initiating in non-coding regions of the bacterial genome.

### Differentiation of ***bona fide*** intragenic transcription start sites (iTSS) from gene background signals

**Fig. 4:**
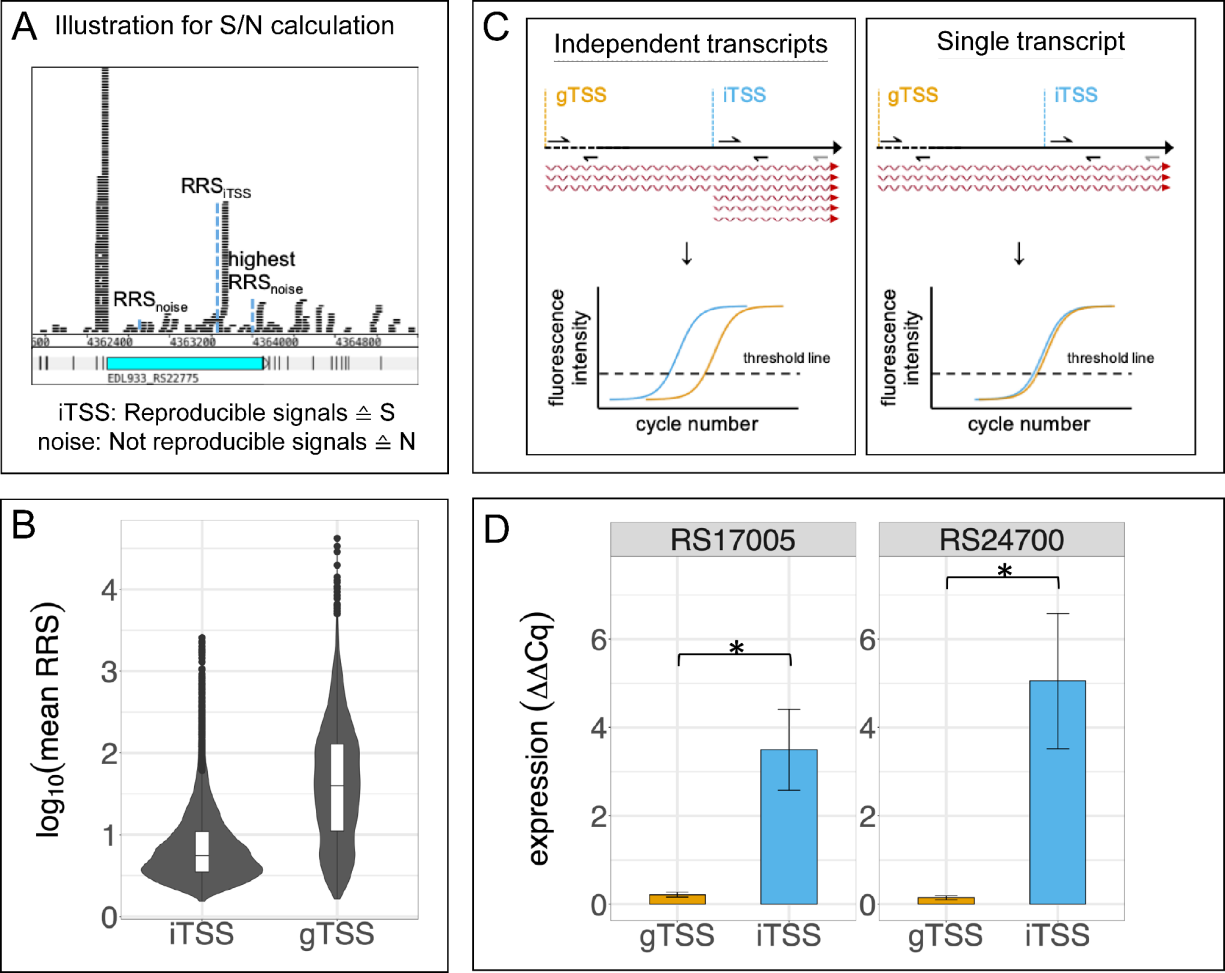
Sense internal TSS. **A** Illustration of the strategy to differentiate genuine internal TSS from background signals. iTSS represents a reproducible internal TSS (= signal = S). The relative read score is compared to the RRS of the position with the highest signal, which is not reproducibly detectable as a TSS in all replicates (= noise = N). iTSS with $\frac{S}{N}>1.5$ in all three replicates are considered as genuine internal TSS. **B** Comparison of the signal strength of iTSSs and gTSSs of the corresponding annotated gene. The log_10_ of the mean RRS of three replicates is shown as box plots within the violin plot. Outliers are indicated with dots. The violin plot visualizes the abundance of TSSs at a specific RRS values. **C** Schematic overview for RT-qPCR quantification of different mRNA molecules of internal and gene associated TSS of an annotated gene. The RT-primer (gray) was used for cDNA synthesis of the respective RNA transcripts. Two primer pairs (black) were designed to amplify ~ 100 bp fragments downstream of the gTSS but upstream of the iTSS (orange) and downstream of the iTSS (blue), respectively, with equal efficiencies. A lowered Cq value in RT-qPCR (earlier crossing with the threshold line) for the iTSS values is an indication for an individual transcript additionally originating from the iTSS as secondary, short mRNA transcripts (left panel). If the same mRNA is used for amplification of gTSS and iTSS fragments (i.e., iTSS is not present or weak), similar Cq values are expected (right panel). **D** Quantification of mRNA originating from transcripts starting at gTSS and iTSS. Two genes, EDL933_RS17005 and EDL933_RS24700, are shown here. The normalized expression (ΔΔCq) regarding the gene *cysG* in LB medium (exponential phase) is used as normalizing gene. Mean value and standard deviation was calculated based on three biological replicates for the two genes indicated. Statistical significance between the normalized expression was verified with a one-tailed Welch two sample t-test (* p ≤ 0.05)

Another class of non-canonical TSS can be observed same strand within annotated genes, intragenic TSS (iTSS). To differentiate background signals originating from annotated genes and *bona fide* iTSS, the signal strength of potential iTSS within annotated genes at cutoff 1.5 were compared to the highest background signal originating from the corresponding annotated gene, i.e. the highest non-reproducible signals (illustrated in Fig. 4A). The ratio between a potential iTSS signal and background noise (S/N) was calculated for all three replicates in the condition where the signal is present. The proportion indicates whether the TSS is less or equally (S/N ≤ 1) or higher (S/N > 1) expressed than the background arising from annotated genes. Only positions with an increased S/N ratio of at least 1.5 in all three replicates were considered as potential internal TSS.

With these criteria, 4,637 TSS with an increased expression (i.e, S/N > 1.5) were identified (Supplementary Table S7). However, 1,233 TSS may belong to annotated genes downstream while their TSSs are located within the upstream gene, or the TSS is downstream of the falsely annotated start codon of the particular annotated genes as shown above. In any case, these gene-associated TSS with increased expression are predominantly localized at the 3’ or 5’ ends of the respective annotated genes, but less often in the middle of the gene in question (Supplementary Figure S2A). Finally, 73% of the TSS (3,404) are not associated to the annotated gene in which they appear. Thus, these iTSSs may represent stable iTSSs independent of surrounding annotated genes, as they are more evenly distributed over the annotated gene length (Supplementary Figure S2B). Nevertheless, despite increased expression compared to the background, the overall strength of the iTSS tends to be lower compared to the main TSS of the corresponding annotated genes (Fig. 4B).

As a test case, a RT-qPCR was performed to investigate the presence of independent mRNA molecules produced from internal transcription start sites (Fig. 4C, Supplementary Table S8). We analyzed the genes for recombination-promoting nuclease B (EDL933_RS17005) and a lipoprotein, which is conserved among Enterobacteriaceae (EDL933_RS24700). Both annotated genes have a main TSS (76 bp and 28 bp upstream of the start codon at genome positions 3236791 and 4749267, respectively) and an internal TSS at genome positions 3236065 (650 bp downstream of start codon) and 4749647 (352 bp downstream of start codon), respectively. The relative read scores of the iTSSs exceed the RRSs of the gTSS in the condition analyzed (exponential phase, LB) and S/N is greater than 6, revealing clear above background transcription. Primers for RT-qPCR were designed to amplify an approximately 100-bp fragment downstream of each transcription start site at similar efficiencies to ensure unbiased relative quantification (Fig. 4C). The normalized expression is significantly higher for both iTSS amplicons compared to the gTSS amplicons indicating an increased amount of mRNA produced from the internal TSS (Fig. 4D). These data support the hypothesis of truly independent iTSSs producing shorter mRNAs in addition to the long RNAs starting at the upstream TSSs.

### Promoters of canonical and non-canonical TSS

**Fig. 5:**
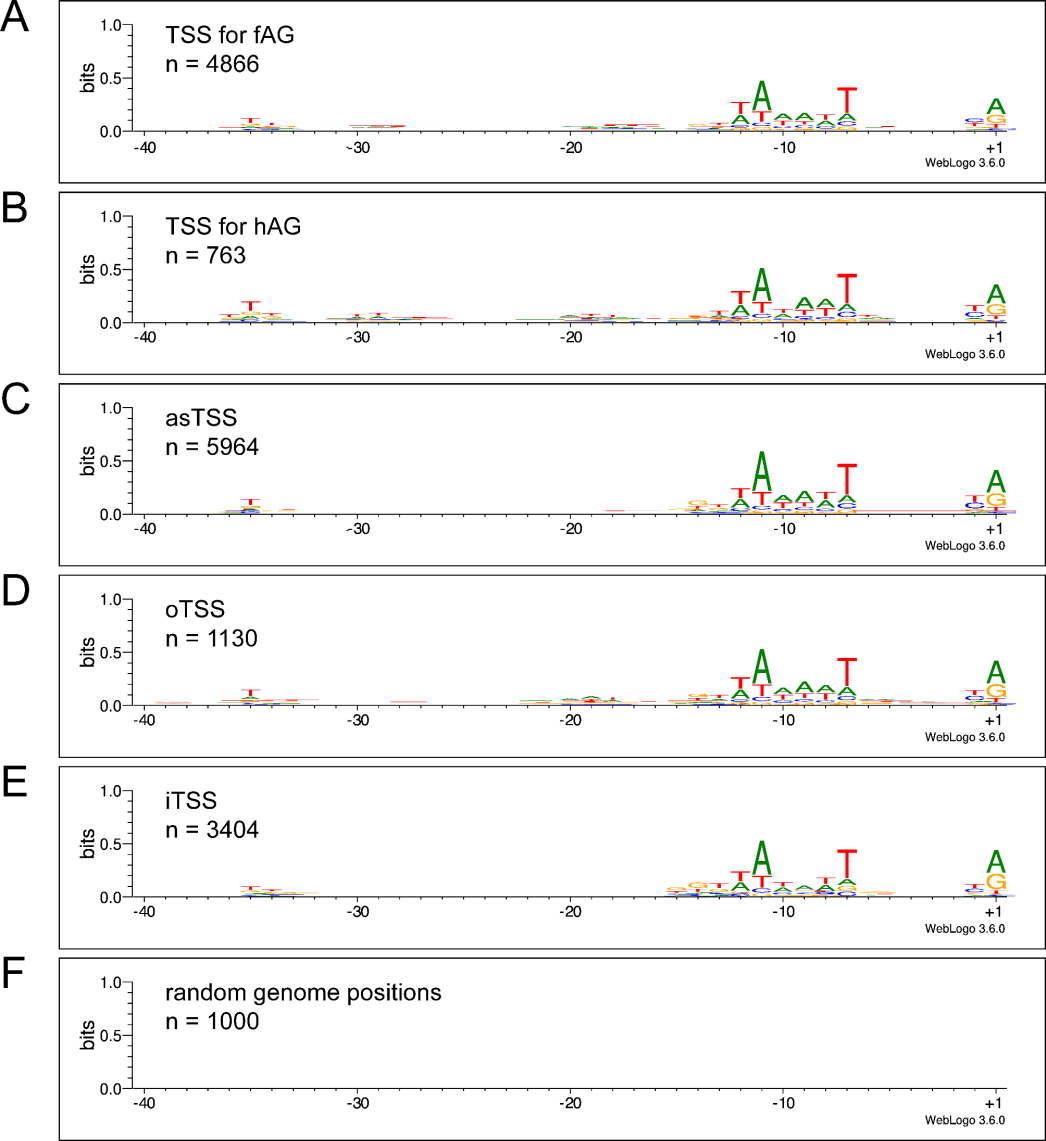
Promoter conservation represented as sequence logos. Regions upstream of gTSS for functional annotated genes (fAG, **A**), hypothetical annotated genes (hAG, **B**), asTSS (**C**), oTSS (**D**), iTSS (**E**), and random genome positions (**F**) are shown

We analyzed the sequences upstream of each TSS category to search for characteristic structures of bacterial promoters. A highly conserved − 10 region (Pribnow box) and the less conserved − 35 region was found for canonical as well as non-canonical TSS (Fig. 5A-E). In contrast, for a number of random genome positions no sequence pattern was detected (Fig. 5F), which strengthens the conclusion that TSSs detected with Cappable-seq are specific and are start points for targeted expression of canonical as well as non-canonical transcripts.

To verify the finding that non-canonical TSSs are indeed active sites, we conducted in-vitro promoter assays using five asTSS and one oTSS (Supplementary Figure S3), spread out on the entire genome, at positions 300490, 1985981, 2285574, 2285499, 3226912, and 4867698 (oTSS), respectively. Two asTSS were chosen in close proximity: asTSS 2285574 and 2285499 are 75 bp apart. Interestingly, despite the low distances between the two particular asTSSs, the programs bTSSfinder and BPROM predicted different promoters for each of the TSS and specific activities were verified for these (Supplementary Figure S3).

We further focused on the promoter-prediction program bTSSfinder for high-throughput analysis of promoters upstream of the 5,567 canonical and 10,352 non-canonical TSSs detected via Cappable-Seq. Thereof, 4,941 (89%) and 8,554 (83%), respectively, were predicted to have a sigma factor specific promoter (Supplementary Table S9). In contrast, bTSSfinder predicted for approximately 30% of 1000 random genome positions a promoter. Next, sequence logos were prepared for canonical as well as non-canonical TSSs. Clear promoter motifs were found for those TSS which had a predicted promoter in the previous step (Supplementary Figures S4A-B), whereas random genome positions did not show any conserved pattern (Supplementary Figure S4C). Unexpectedly but strikingly, upstream sequences of non-canonical TSS without any promoter prediction in the previous step showed a highly conserved adenine at position − 10 and a thymine at position − 7 (Supplementary Figure S4B, right panel).

### Differential expression of canonical and non-canonical TSS

As described above, TSSs were determined in four different culture conditions, each at exponential growth and early stationary growth, in biological triplicates. Based on this large data set, we were able to analyze differential expression of canonical and *bona-fide* non-canonical TSS using the *Bioconductor* package *edgeR*. We analyzed condition specific (stress condition compared to LB medium) and growth phase dependent differentially expressed TSS (FDR < 0.05, log_2_FC > |2|; Supplementary Table S10).

**Fig. 6:**
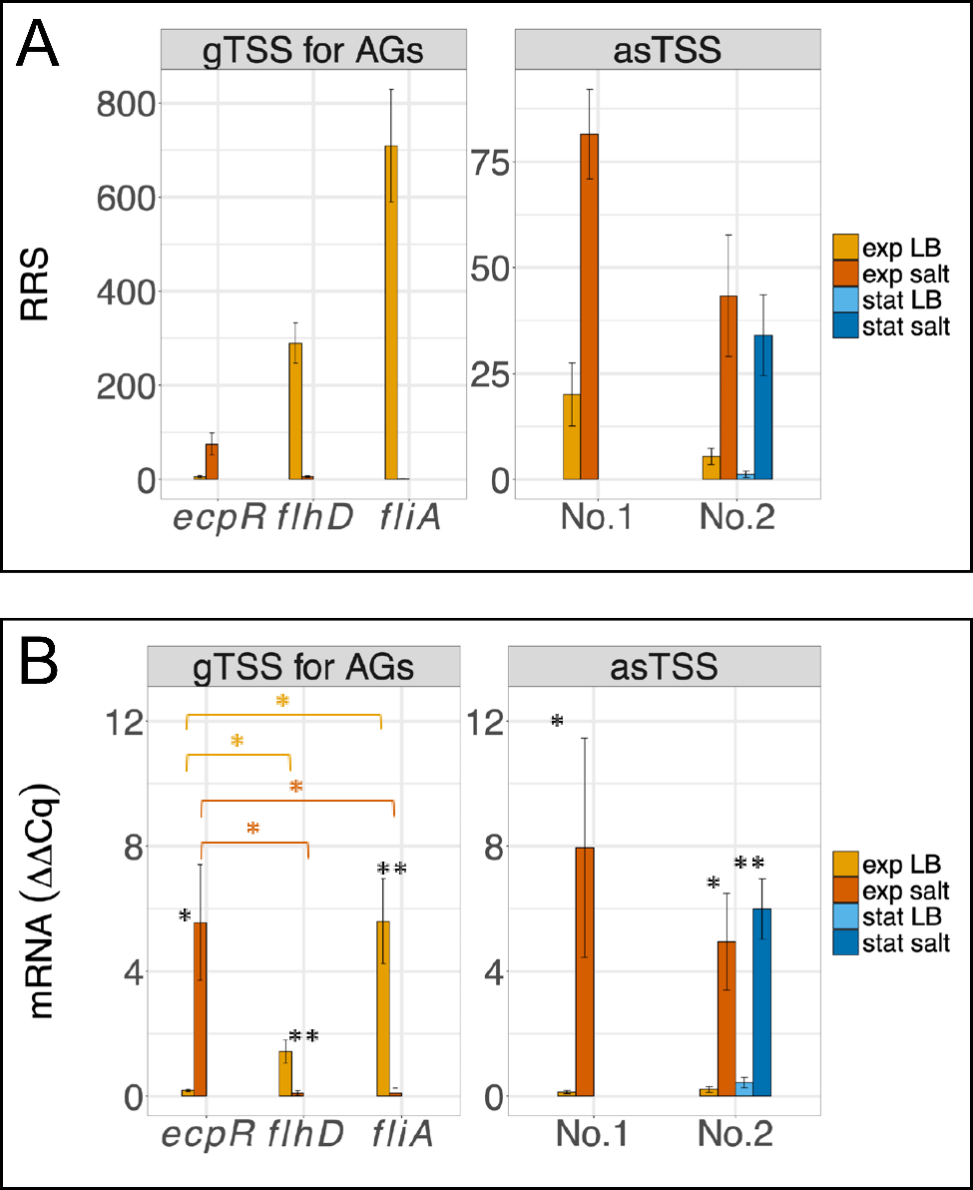
Verification of differential TSS expression. **A** Relative read scores (RRS) for TSS of annotated genes (*ecpR*, *flhD*, *fliA*) and asTSS at genome positions 4,763,189 (asTSS No. 1) and 2,742,524 (asTSS No. 2) from Cappable-seq libraries. Mean RRS of three biological replicates and the standard deviation are given. Differential expression was statistically verified at FDR < 0.05 and log_2_ FC >|2| between LB and salt supplemented LB in all instances. Orange, LB in exponential phase; red, LB + salt in exponential phase; light blue, LB in stationary phase; dark blue, LB + salt in stationary phase. **B** Normalized expression (ΔΔCq) of *ecpR*, *flhD*, *fliA*, and of asTSS No. 1 and asTSS No. 2 according to Fig. 6A. Quantification was performed with RT-qPCR. Expression of the gene *cysG* (siroheme synthase) was used for normalization. Significant up- or down-regulation in salt-supplemented medium was statistically significant for *ecpR*, asTSS No. 1, and asTSS No. 2 or *flhD* and *fliA*, respectively (one-tailed paired t-test, * p ≤ 0.05; ** p ≤ 0.01). Orange, LB in exponential phase; red, LB + salt in exponential phase; light blue, LB in stationary phase; dark blue, LB + salt in stationary phase

The accuracy concerning the determination of differential TSSs expression was analyzed using two well-known test cases, the LEE pathogenicity island and genes for flagellum synthesis. Both genetic islands have been examined in detail in the past and gene expression data are available [64, 65]. The main TSSs for ten of eleven operon elements of the LEE pathogenicity island were identified [Supplementary Figure S5A, 66]. In our data, expression of these elements is continuously upregulated in exponential growth phase when comparing minimal medium to LB medium (Supplementary Figures S5B-C), but we found most TSSs are less expressed in minimal medium in stationary phase compared to LB medium. This is in line with observations of TM Bergholz, LM Wick, W Qi, JT Riordan, LM Ouellette and TS Whittam [64] and N Nakanishi, H Abe, Y Ogura, T Hayashi, K Tashiro, S Kuhara, N Sugimoto and T Tobe [65]. Concerning the second example, a RT-qPCR was performed for three genes involved in the regulation of flagellum synthesis (EDL933_RS01675, *ecpR*; EDL933_14025, *flhD*; and EDL933_RS14325, *fliA*). This allowed comparing differential TSS signals from the Cappable-seq experiment (Fig. 6A) with the actual gene expression (i.e., amount of mRNA produced; Fig. 6B, Supplementary Table S8). Indeed, elevated levels of *flhD* and *fliA* in LB medium compared to *ecpR* expected from the TSS-data were confirmed with the RT-qPCR (one-sided Welch two-sample t-test, p-values < 0.05) as well as increased expression of *ecpR* during salt stress compared to *flhD* and *fliA* (one-sided Welch two-sample t-test, p-values < 0.05). Additionally, differential TSS expression for two asTSS (No. 1, asTSS 4,763,189; No. 2, asTSS 2,742,524) was verified with RT-qPCR (Fig. 6A-B). In summary, differential expression signals detected with Cappable-seq are reproducible and can be verified for canonical as well as non-canonical TSS. Such differential expression, as discussed below, can be interpreted as evidence for regulation.

**Fig. 7:**
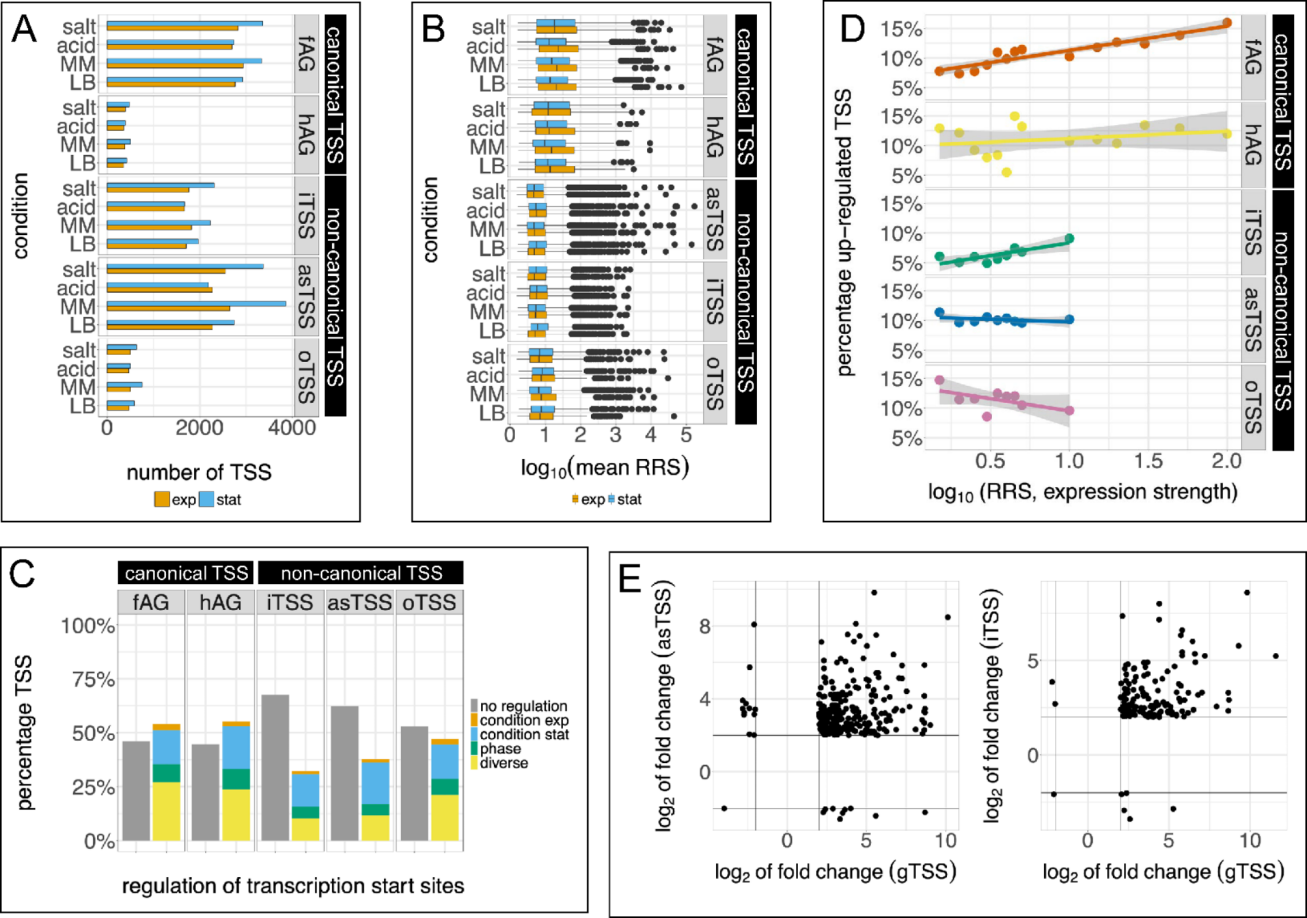
Differential expression patterns of canonical and non-canonical TSS. **A** Number of reliably identified TSS in exponential (exp, orange) and stationary (stat, blue) growth phases visualized for the different culture conditions: LB medium supplemented with 500 mM NaCl (salt), LB medium supplemented with 4 mM L-malic acid (acid), minimal medium (MM), and plain LB medium. Number of TSS are shown for canonical TSS for annotated genes associated with a function (fAG, n = 4866), annotated hypothetical genes (hAG, n = 763), and non-canonical TSS (iTSS, n = 3404; asTSS, n = 5967; oTSS, n = 1130). **B** Visualization of expression strength (log_10_ of the mean RRS of three replicates) of transcription start sites of different gene groups in different experimental conditions, as indicated, respectively. **C** Differentially regulated TSS. Percentage of TSS found not regulated (gray) and regulated (colored) in our experiments. Orange, regulated in LB compared to LB / salt, LB / acid, and minimal medium in exponential phase; blue, regulated in LB compared to LB / salt, LB / acid, and minimal medium in stationary phase; green, regulated between growth phases; yellow, miscellaneous regulation patterns not restricted to either growth conditions or growth phases. **D** Regulated TSS depending on expression strength (x-axis). Percentage of up-regulated TSS are shown for canonical (gTSS of functional and hypothetical annotated genes: fAG, hAG) and non-canonical TSS (iTSS, asTSS, oTSS). Expression strength classification was adjusted for canonical and non-canonical TSS according the overall expression strength of the TSS as shown in (**B**). RRS expression strength categories were defined as follows: RRS 1.5-2, 2-2.5, 2.5-3, 3-3.5, 3.5-4, 4-4.5, 4.5-5, 5–10, 10–15, 15–20, 20–30, 30–50, 50–100, > 100. **E** Comparison of significant regulation of non-canonical asTSS/iTSS in relation to the corresponding canonical gTSS of each annotated gene. Log_2_(fold change) values of gTSS (x-axis), asTSS (y-axis, left panel) and iTSS (y-axis, right panel) are given. Only data points with significant fold changes of |2|, indicated with black lines, are shown

To obtain more insight in the different groups of TSSs, we compared the expression and regulation of canonical TSS and non-canonical TSS in more detail. We had detected 4,866 canonical gTSS in functional annotated genes, and 763 gTSS for hypothetical annotated genes. Concerning non-canonical, we detected 3,404 iTSS, 5,967 asTSS, and 1,130 oTSS in our experiments. In general, more TSS showed expression in stationary compared to the exponential growth phase (Fig. 7A). Furthermore, on average, 50% of the genes possessing a canonical TSS also have an iTSS. Even more, little more than 50% of the genes exhibit an asTSS. Both findings indicate a genome-wide and unexpected abundance of non-canonical TSS. Globally, the expression strength of the (proper) gTSSs is highest, independent of the annotation status of the corresponding gene (i.e., functional or hypothetical). In contrast, the median expression of non-canonical TSS is approximately one log unit less (i.e., 1/10) compared to the gTSSs, again independently of functional or hypothetical annotated genes (Fig. 7B). Additionally, although more TSS (in total numbers) are observed in stationary phase in almost all experiments (Fig. 7A), the expression strength of the TSSs is elevated in exponential growth phase in most conditions analyzed for canonical TSS, in contrast to non-canonical TSS, where differences can barely be detected between growth phases. We find that significantly more canonical TSS compared to the non-canonical iTSS are regulated. (Fig. 7C). Most TSS are either differentially expressed only between growth conditions in stationary phase (blue, Fig. 7C) or are variously differentially expressed (yellow, Fig. 7C). A considerable number of canonical TSS appear not to be regulated (log_2_FC > |2| and FDR cutoff > -log_10_(0.05), which is most likely due to the limited number of conditions analyzed. However, regardless of the TSS type and regulation category, TSS are more often upregulated than downregulated in all instances compared to non-stress LB (Supplementary Figure S6). Next, we then correlated up-regulated TSS with their expression strength in the regulated stress condition (Fig. 7D). For canonical TSS, especially those of functional annotated genes, we find a higher fraction of regulated TSS in strong expression categories (p < 0.05). For iTSS, a similar trend can be found, whereas for remaining non-canonical TSS (oTSS and asTSS) the numbers of regulated TSS per expression class seem to be inversely correlated (Fig. 7D), although weakly (not significant).

Finally, the fold changes of stress-regulated iTSS and asTSS were compared to significantly expressed TSS (log_2_FC > |2| and FDR cutoff > -log_10_(0.05) of the related annotated genes (Fig. 7E). Similar to previous analyses, most regulation patterns indicate upregulated expression for both, annotated genes and non-canonical transcripts under stress. In order to reveal a possible functional coupling between canonical and non-canonical TSSs of the respective gene, the linear correlation coefficient was calculated for the TSS pairs. However, no correlation was detected for the fold change (asTSS – gTSS, *r* = 0.2; iTSS – gTSS, *r* = 0.27) or the respective mean expression (relative read scores, asTSS – gTSS, *r* = 0.025; iTSS – gTSS, *r* = -0.002). Thus, the data does not provide evidence for a general functional coupling of the gTSS and the associated non-canonical asTSS/iTSS.

## Discussion

The presence of antisense transcription as well as internal and ‘new’ transcription start sites was reported previously for various bacteria [e. g., 1, 2, 4]. However, the current conceptual framework assumes that the majority of such non-canonical start sites and the resulting transcripts are non-functional. They are assumed to be either products of experimental noise or the RNA-polymerase’s pervasive activity, especially if these signals are comparatively weak [16–18, 67, 68]. Indeed, many of the non-canonical TSSs reported here produce weaker signals compared to canonical TSSs (Fig. 7B). Despite increasing evidence, as also presented here, only few authors currently support the notion that a significant number of the non-canonical transcripts may be functional in bacteria [17, 69–71]. While pervasive transcription certainly exists in biological systems, we find a large number of unexpected non-canonical TSSs (i.e., 10,355) with high confidence to be reproducibly active and regulated. Thus, we believe that a greater number of these non-canonical TSSs may be functional than assumed so far.

### Reliability of TSS of the primary EHEC transcriptome

The genome of EHEC is substantially larger and harbors more genes than the genome of the apathogenic *E. coli* MG1655 [~ 5500 genes in 5.5 Mb compared to ~ 4200 genes in 4.6 Mb, 72, 73, 74]. Indeed, more TSS were identified for EHEC [~ 19.000 compared to ~ 16.000 for *E. coli* MG1655, 2, 23]. In our data, the precision of calling a TSS site using the Ettwiller algorithm was ensured by a highly stringent selection criterion (see below). Furthermore, we compared TSSs from homologous genes in *E. coli* MG1655 (Fig. 2) and find substantial overlap in the TSSs position for both strains. In addition, background signals were integrated in our evaluation procedure to distinguish between possible mRNA degradation products and *bona-fide* signals more precisely (Fig. 4).

With respect to the number of annotated genes with a TSS, it may seem unexpected that, despite the analysis of eight substantially different environmental conditions, the percentage of genes with a TSS (54%) is only slightly higher or even less compared to other organisms. In *E. coli* MG1655, 63% of annotated genes have been reported to show a TSS [2]. *Helicobacter pylori* has 812 TSS for 1576 genes [51%, 1], approximately 46% of genes in *Haloferax volcanii* have a TSS [3] and K Papenfort, KU Förstner, J-P Cong, CM Sharma and BL Bassler [75] reported 1831 primary TSS for 3654 coding genes in *Vibrio cholerae* (50%). Perhaps this is due to our selection criterion for TSSs being much more stringent than those in other studies, since we accepted a TSS only if it was consistently supported by three biological replicates. The fact that we detected only slightly more TSSs than have been found in other bacteria, in our opinion, supports the reliability of the signals reported in our study.

Reproducibility of a TSS signal is a prerequisite, but by no means a conclusive argument, for functionality since an RNA polymerase binding sequence may well originate by chance in random AT rich nucleotide sequences [76–78]. Due to the degeneracy of the genetic code, there is little reason to assume that RNA polymerase binding sites should not occur within nucleotide sequences that encode a functional amino acid sequence. Such polymerase binding sequences may also result in a reproducible TSS signal and, hence, in pervasive, nonfunctional transcription. On the other hand, C Mejía-Almonte, SJ Busby, JT Wade, J van Helden, AP Arkin, GD Stormo, K Eilbeck, BO Palsson, JE Galagan and J Collado-Vides [79] noted that a standard promoter is essential for transcription initiation at specific transcription start sites.

### Do standard promoter motifs upstream of non-canonical TSS indicate functionality?

We investigated the sequences upstream of reliable TSS for the presence of conserved promoter sequence motifs. Not surprising, annotated genes including hypothetical genes yielded a clear standard − 10 and a weak − 35 and clear − 1/+1 promoter motif. Since the motif shown in Fig. 5A reflects the average of 4,866 annotated sequences carrying all sorts of different promoters, and since it is still not possible to accurately predict whether a DNA sequence harbors a promoter [80], the average motif is 0.7 bits at maximum. This value is clearly less than the one observed for a motif of, e.g., a small number of well-characterized σ^70^ promoters [> 1.2 bits; 81]. Very surprisingly, however, non-canonical TSS (Fig. 5C-E) showed a virtually identical overall standard promoter motif compared to that of annotated genes (Fig. 5A-B). In contrast, random genome positions did not yield any motif at all (Fig. 5F).

Experimental evidence has been presented that weak RNA polymerase binding sites can evolve easily to standard promoter sequences if a positive selection pressure is applied [76, 82]. However, this process should require some sort of functionality of the transcripts produced. We find it difficult to understand why over-all promoter motifs initiating non-functional pervasive transcription should have evolved an almost perfect identity with evolutionarily highly optimized promoter motifs of the functional, annotated genes of the cell.

Due to the limited information content of promoters [e. g., 77, 83], in some cases sequences similar to standard promoters will occur by chance without evolutionary optimization, leading to pervasive transcription. Pervasive transcription is associated with an energetic cost, which would be correlated to the fraction of pervasive transcription. If that fraction is large enough, a fitness cost for the cells would be expected which impairs cellular functions [84]. V Lloréns-Rico, J Cano, T Kamminga, R Gil, A Latorre, W-H Chen, P Bork, JI Glass, L Serrano and M Lluch-Senar [16], based on theoretical analyses, suggested that the energetic impact of spurious transcription is very low. Our data indicate that about 35% of the total reads associated to high-confidence TSS in EHEC under the conditions analyzed belong to non-canonical TSS. While this data does not allow to estimate an energetic cost, which is related to the fraction of all non-functional RNA reads, TY Michaelsen, J Brandt, CM Singleton, RH Kirkegaard, J Wiesinger, N Segata and M Albertsen [70] reported that between approx. 4 to 50% of all genes in the metagenomes of five different, complex microbial communities produce antisense reads. Such numbers would imply an energetic cost for the cell, leading to purifying selection and, as a consequence, the disappearance of promoter motifs, which initiate pervasive transcription. Indeed, it was shown recently that a strong selection acts against promoter motifs in *E. coli* [76, 78] and the introduction of AT-rich DNA by horizontal gene transfer is toxic for the cell due to sequestering RNA polymerase [85]. Interestingly, it has been speculated that there may be a preferential codon usage in protein coding genes to avoid promoter-like sequences [80].

A further line of evidence, which implies functionality of non-canonical TSS, is evolutionary conservation across species, which indicates purifying selection. W Shao, MN Price, AM Deutschbauer, MF Romine and AP Arkin [86] reported about 30% of iTSS and 22% of asTSS being conserved between 8 *Shewanella* species, while 19% of asTSS were conserved between two *Halobacterium* species [4].

### Differential expression of TSS indicates gene regulation

One approach to identify differentially regulated TSS is the analysis of high-confidence TSS under a number of different stress conditions and growth phases. In order to investigate this approach for our Cappable-Seq data sets in some test cases, the regulation of several genes reported in the literature were analyzed. Three TSS signals of *ecpR*, *flhD*, and *fliA*, involved in the regulation of flagellum synthesis [87–90], and which were also seen in our Cappable-Seq data were examined using RT-PCR (Fig. 6). Our results demonstrate that differential expression profiles derived from our Cappable-Seq data set TSS are similar to known ones and can be used to determine regulation patterns.

The analysis of differential TSS expression under various environmental conditions yielded many non-canonical TSS for which differential expression was observed. Non-canonical TSS are clearly less expressed (Fig. 7B) and a considerable smaller number (p < 0.05) is differentially expressed (Fig. 7C). The absence of differential expression at non-canonical TSS sites, however, is not necessarily equivalent to non-functionality because almost half of the canonical TSS of annotated genes are not regulated under our conditions as well (Fig. 7C). Differential expression can be evidence for regulation and, thus, for functionality [86]. However, it cannot be excluded that activator or operator motifs also occur by chance in the vicinity of RNA polymerase binding sites since transcription factor binding sites can evolve rapidly via local point mutations [91]. In such cases, differential gene expression would not be indicative for functionality.

## Conclusion

Cappable-seq was performed to determine the primary transcriptome of the human pathogen EHEC for the first time. Based on the reproducible determination and differential expression of canonical and non-canonical TSS, we suggest that a considerable number of non-canonical TSS, while often substantially less expressed than canonical TSS, are functional, rather than constituting pervasive transcription only. We therefore conclude that the EHEC transcriptional landscape is more complex than previously assumed. However, future studies are now required, such as data on transcription stop sites, analysis of regulatory mechanisms for condition-specific transcription of individual non-canonical TSS and their functional characterization, including potential gene expression products. Only then, a more detailed picture of the highly complex transcriptional landscape of the foodborne pathogen EHEC will emerge.

### Electronic supplementary material

Below is the link to the electronic supplementary material.

Supplementary Material 1

Supplementary Material 2

Supplementary Material 3

Supplementary Material 4

Supplementary Material 5

Supplementary Material 6

Supplementary Material 7

Supplementary Material 8

Supplementary Material 9

Supplementary Material 10

Supplementary Material 11

## Data Availability

All raw sequencing data have been deposited at the NCBI Sequence Read Archive (SRA) under the accession numbers SRR24463161 to SRR24463192 (BioProject number PRJNA853291, study number SRP436388, BioSample numbers SAMN34994604 to SAMN34994635).
