# Supplementary material for: Non-canonical transcriptional start sites in E. coli O157:H7 EDL933 are regulated and appear in surprisingly high numbers
Source: BMC Microbiol. 2023 Aug 31;23:243. doi: 10.1186/s12866-023-02988-6 (PMC10469882; doi:10.1186/s12866-023-02988-6)

Supplementary Data

Supplementary File 1 Software tool versions and parameters used.

Supplementary Figure 1 Overview of the applied Cappable-seq workflow of Ettwiller et al. (2016) adapted and conducted by Vertis Biotechnologie AG, Freising.

Supplementary Figure 2 Distribution of internal TSSs over the length of the annotated genes.

Supplementary Figure 3 Promoter activity assay.

Supplementary Figure 4 Sequence logos of canonical TSS, non-canonical TSS and random genome positions with and without predicted promoter sequence.

Supplementary Figure 5 Differential TSS expression for the LEE Pathogenicity Island.

Supplementary Figure 6 Volcano plots for transcription start sites of different categories: gTSS of fAG and hAG, iTSS, asTSS, and oTSS.

Supplementary Table S1 Growth conditions (**A**), optical density (**B**) at the time point of cell harvest, harvested culture volume (**C**) and Trizol volume (**D**) used for RNA isolation.

Supplementary Table S2 Details for primers used in cDNA synthesis and qPCR analysis. Primer name (**A**), sequence (**B**), primer description (**C**), annealing temperature in qPCR (**D**), and primer efficiency (**E**).

Supplementary Table S3 Details for primers used promoter cloning for promoter activity analysis. Primer name (**A**), sequence (**B**), primer description (**C**), length of PCR product (**D**), length of cloned promoter sequence (**E**).

Supplementary Table S4 List of transcription start sites in *E. coli* O157:H7 EDL933 (**A, B**), their presence in analyzed culture conditions (**C-J**), and categorization in gTSS (**K**), subdivided in gTSS for functional annotated genes (fAG, **L**) and hypothetical annotated genes (hAG, **M**), d_gTSS (**N**), iTSS (**O**), asTSS (**P**), oTSS (**Q**). For iTSS, the number of conditions is indicated where the TSS has an increased signal strength. Red shaded TSS are iTSS with insufficient signal strength. The visual assignment of orphan TSS to annotated genes, tRNA-, rRNA-, tmRNA-genes, ncRNA, pseudogenes, and oTSS is indicated in **Q**.

Supplementary Table S5 Gene specific TSS. Detailed lists (sheets 1 and 2) of annotated genes with gTSS and d_gTSS showing identifier, strand and TSS position of the annotated gene (**A-C**), TSS presence in analyzed culture conditions (**D-K**) and gene annotation status (**L**). Additionally, the TSS comparison between EHEC and *E. coli* MG1655 is included (sheet 3) with the name of the gene (gene number for EHEC genes) and the distance between start codon and TSS for MG1655 (**A, B**) and EHEC (**C, D**) and the deviation between MG1655 and EHEC distances (**E**).

Supplementary Table S6 List of annotated genes with antisense TSS. Annotated gene number, strand, start and stop coordinates (**A-D**), asTSS position and strand (**E**, **F**), asTSS presence in analyzed culture conditions (**G-N**), categorization of asTSS in u-asTSS, d-asTSS and asTSS (**O**).

Supplementary Table S7 List of annotated genes with iTSS: annotated gene number (**A**), iTSS position (**B**), absolute distance of iTSS and start codon of annotated gene (**C**), relative position of iTSS within annotated gene (**D**), iTSS abundance (**E**), S/N ratio of the iTSS in the analyzed conditions (**F-M**; dark red shaded positions, no TSS present in this condition; light red shaded positions, S/N ratio smaller than the threshold of 1.5), mean relative read score of the iTSS in the analyzed conditions calculated for three biological replicates (**N-U**), main TSS position (**V-**

AC) and the mean relative read scores (**AD-AK**) for the annotated genes in the conditions analyzed.

Supplementary Table S8 qPCR Cq raw values for candidates and conditions analyzed (**A, B**). Primer efficiencies (**C**), mean quantification cycles of three technical replicates (**D**, mean Cq), Cq of NRT control (**E,** no reverse transcriptase control), mean Cq value for the sample with the lowest Cq for the gene of interest (**F**, $Cq_{(min)}$), ΔCq (**G**), ΔΔCq regarding the reference gene *cysG* (**H**), mean ΔΔCq (**I**) and standard deviation (**J**) of three biological replicates. Missing detection of a PCR product via fluorescence is indicated with NA ('not available').

Supplementary Table S9 Promoter prediction for canonical TSS and non-canonical TSS. The output of the bTSSfinder promoter prediction is given for promoters of canonical TSS (first sheet), promoters of non-canonical TSS (second sheet), and promoters of random genome positions (third sheet).

Supplementary Table S10 Differentially expressed TSS sorted into lists of differentially expressed TSS between LB and minimal medium, LB + acid, or LB + NaCl in exponential growth phase (sheet 1), or in stationary growth phase (sheet 2) or between growth phases (sheet 3). TSS are listed according their TSS category (TSS for fAG or hAG, iTSS, asTSS, oTSS). Each list shows the TSS position (**A**) and strand (**B**), TSS category (**C**), $\log_2$(FC) for different comparisons (upregulated TSS are marked in green, downregulated TSS in yellow, **D-F/D**), p-value (**G/E**), false discovery rate (FDR, corrected p-value after Benjamini-Hochberg, **H/F**), and overall regulation (**I/G**).

Supplementary File S1:

Tool version numbers, settings and input file instructions for programs used in Cappable-seq sequencing evaluation and data evaluation.

Cutadapt

```
cutadapt -u 2 -g ^CGCTCATT -g ^GAGATTCC -e 0.14 -O 8 --no-indels -o
OUTPUT.fastq --untrimmed-output untrimmed_file.fastq INPUT.fastq
```

```
cutadapt -u 2 -o INPUT.fastq OUTPUT.fastq
```

Trimmomatic

Version number: 0.36

input file: PSS–tag/TSS–tag reads in separate fastq-files

output file: trimmed fastq-files

quality trimming file adapter.fasta:

PolyA80 (A)80

PolyT80 (T)80

PolyG80 (G)80

PolyAG AAAAAAAAAGGGAGGGGGGGGGGGGG

```
java –jar trimmomatic–0.36.jar SE INPUT.fastq OUTPUT_trimmed.fastq
-phred33 ILLUMINACLIP:adapter.fasta:2:30:10 SLIDINGWINDOW:4:15
LEADING:3 TRAILING:3 MINLEN:30
```

Bowtie2

Version number: 2.2.9

input file: quality trimmed reads in fastq-files

output file: mapped reads in .sam-file

```
bowtie2 --local –q –p 16 –x genome –U INPUT.fastq –S mapped.sam
```

samtools

version number: 1.9

input file: mapped reads in .sam-file

output file: mapped reads in .bam-file

```
samtools view –Sb mapped.sam > mapped.bam
```

input file: mapped reads in .bam-file

output files: mapped reads sorted and indexed in .bam-file and .bai-file for read visualization

```
samtools sort mapped.bam –o sorted.bam
samtools index sorted.bam sorted.bam.bai
```

bam2firstbasegtf.pl

input file: sorted .bam-file

output file: enriched TSS positions in .gtf-file

```
bam2firstbasegtf.pl --bam sorted.bam --cutoff 1.5 --lib_type F --out
TSS_enriched.gtf
```

cluster_tss.pl

input file: output of bam2firstbasegtf.pl in gtf format

output file: clustered TSS positions in .gtf-file

```
cluster_tss.pl --tss TSS_enriched.gtf --cutoff 5 --out clustered.gtf
```

Weblogo

Version number 3.6.0

input file: sequences for sequence logo creation in fasta-format

output file: sequence logo in pdf-format

```
weblogo -l -50 -u 0 -n 51 --first-index -100 --yaxis 1 --errorbars No -
-format pdf --color-scheme classic --number-interval 10 < input.fasta >
output.pdf
```

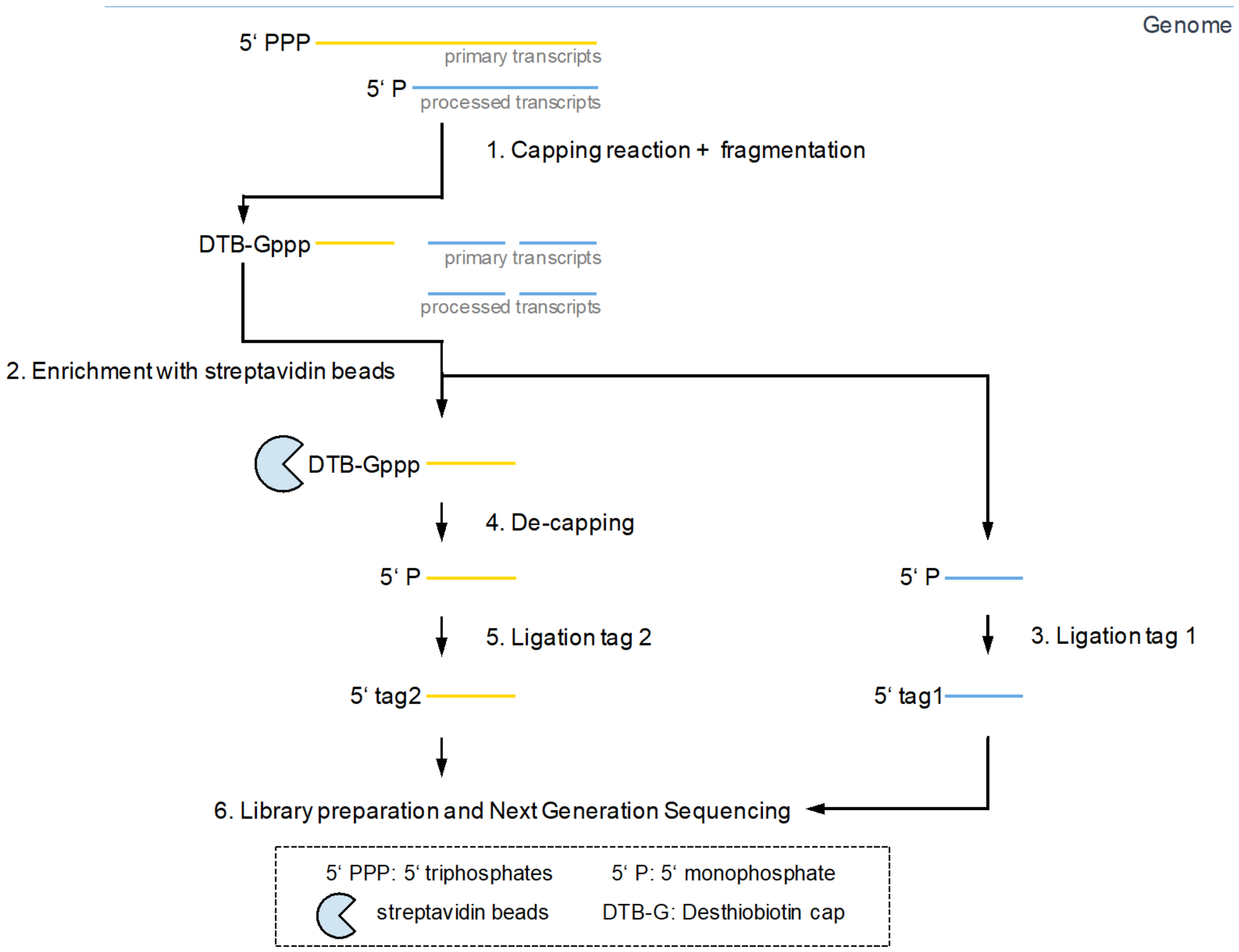

Supplementary Figure S1 Overview of the applied Cappable-seq workflow of Ettwiller et al. (2016) adapted and conducted by Vertis Biotechnologie AG, Freising.

1, Primary transcripts with 5' triphosphates (yellow) are labeled with a desthiobition cap (DTG-TEG-GTP) at the 5' end using vaccinia capping enzyme, whereas fragments with 5' monophosphates (blue) remain unchanged.

2, After fragmentation, 5' monophosphorylated fragmentation products are left unmodified, while biotinylated 5' fragments are captured with streptavidin beads.

3, Contaminating monophosphorylated fragments are ligated to a 5' Illumina TruSeq sequencing adapter with unique sequence tag 1.

4, The desthiobiotin cap is removed using a Cap-Clip Acid Pyrophosphatase and

5, fragments are ligated to 5' Illumina TruSeq sequencing adapter with unique sequence tag 2.

6, Fragments are then used to create a Next Generation Sequencing library. Libraries were sequenced single end (75 bp) with an Illumina NextSeq 500.

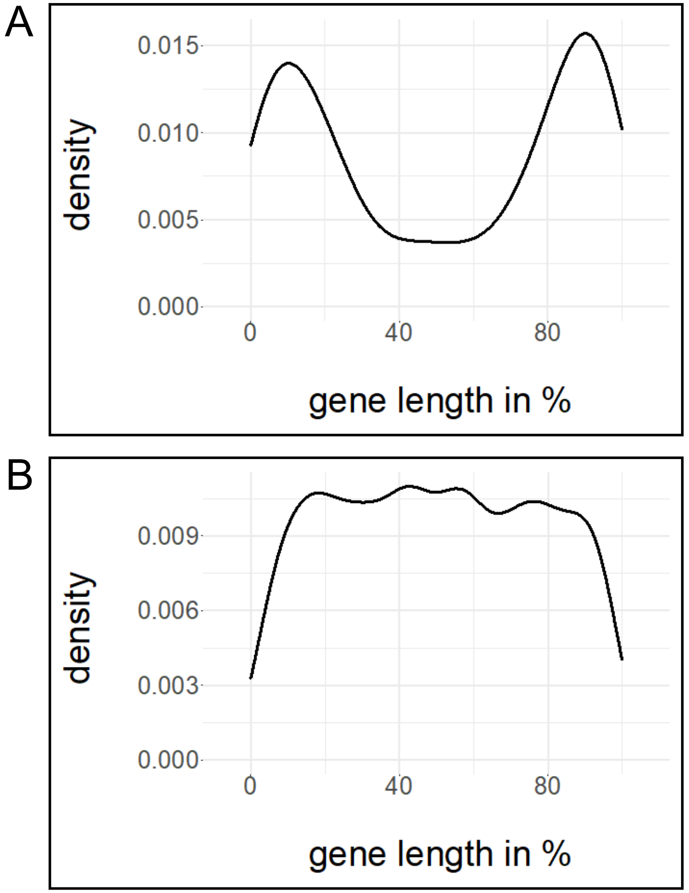

Supplementary Figure S2 Distribution of internal TSSs over the length of the annotated genes. Number of iTSSs are represented as density distribution depending on their localization within the length-normalized annotated gene.
(**A**) iTSSs associated with annotated genes (i.e., 1,233 TSS which may belong to the next gene downstream or belong to annotated genes that have a wrongly annotated start codon and (**B**) genuine iTSSs (i.e., 3,404 TSS with an increased S/N ratio but not classified to belong to the AG).

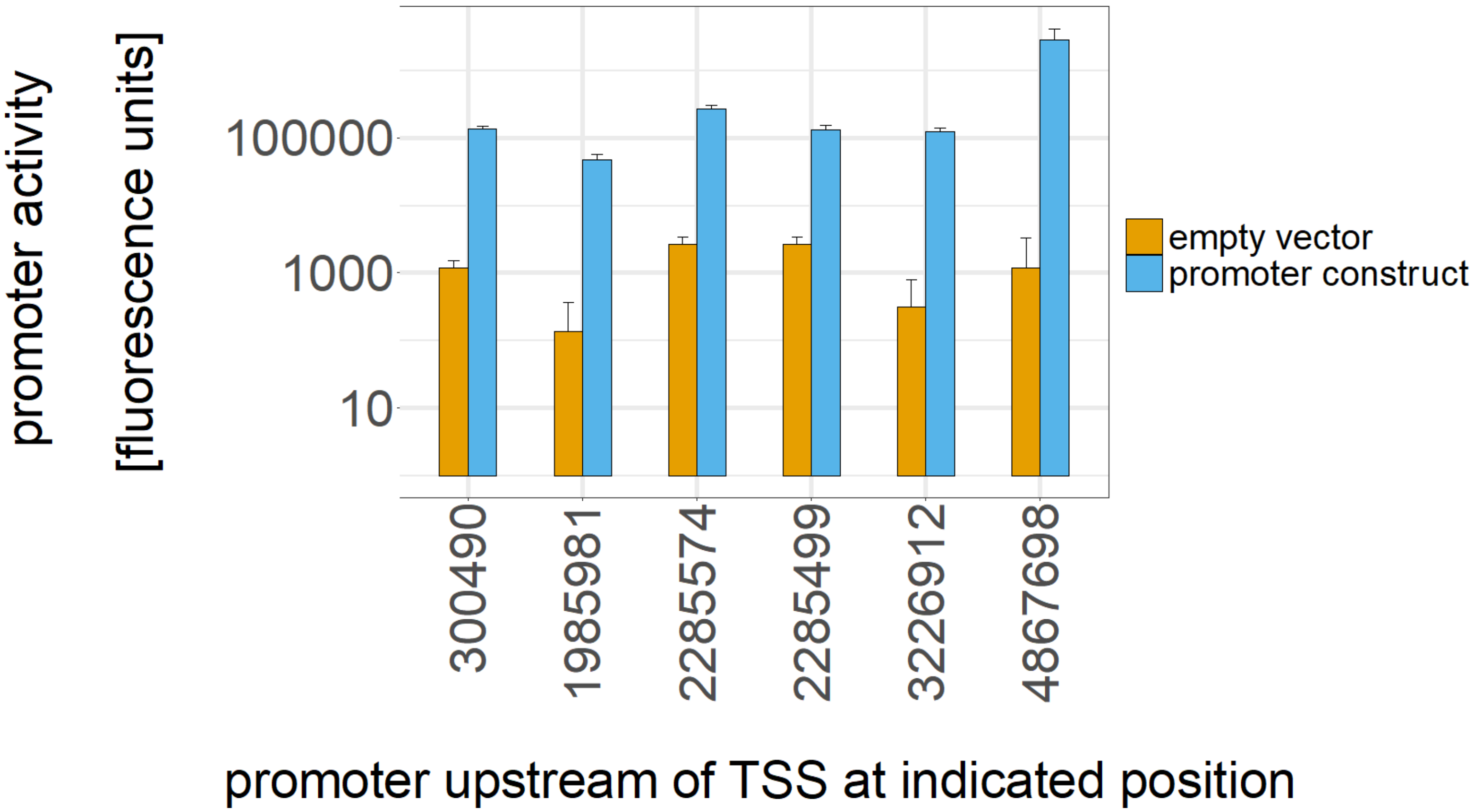

Supplementary Figure S3 In-vitro activity assay of different promoters (positions of the TSS is indicated below the graph). Promoters for five asTSSs and one oTSS (position 4867698) were identified. Promoter sequences (i.e., fragments upstream of each TSS) were introduced in the promoterless GFP reporter vector pProbe-NT and the construct was transformed in *E. coli* Top10. Cells were grown in LB medium supplemented with 450 mM NaCl (for promoter constructs at TSS 2285574 and 2285499) or plain LB medium (all other constructs) until $OD_{600} = 0.6$ was reached and fluorescence was measured. Promoter activity is shown as mean fluorescence of three biological replicates for cells grown with the vector-promoter construct (blue bars) or with the empty vector (orange bars). The latter represents the background signal of the vector in the respective experiment. Significant enhanced activity for analyzed promoters was identified in all instances ($p < 0.05$, Welch two-sample t-test, significance level $\alpha = 5$ %).

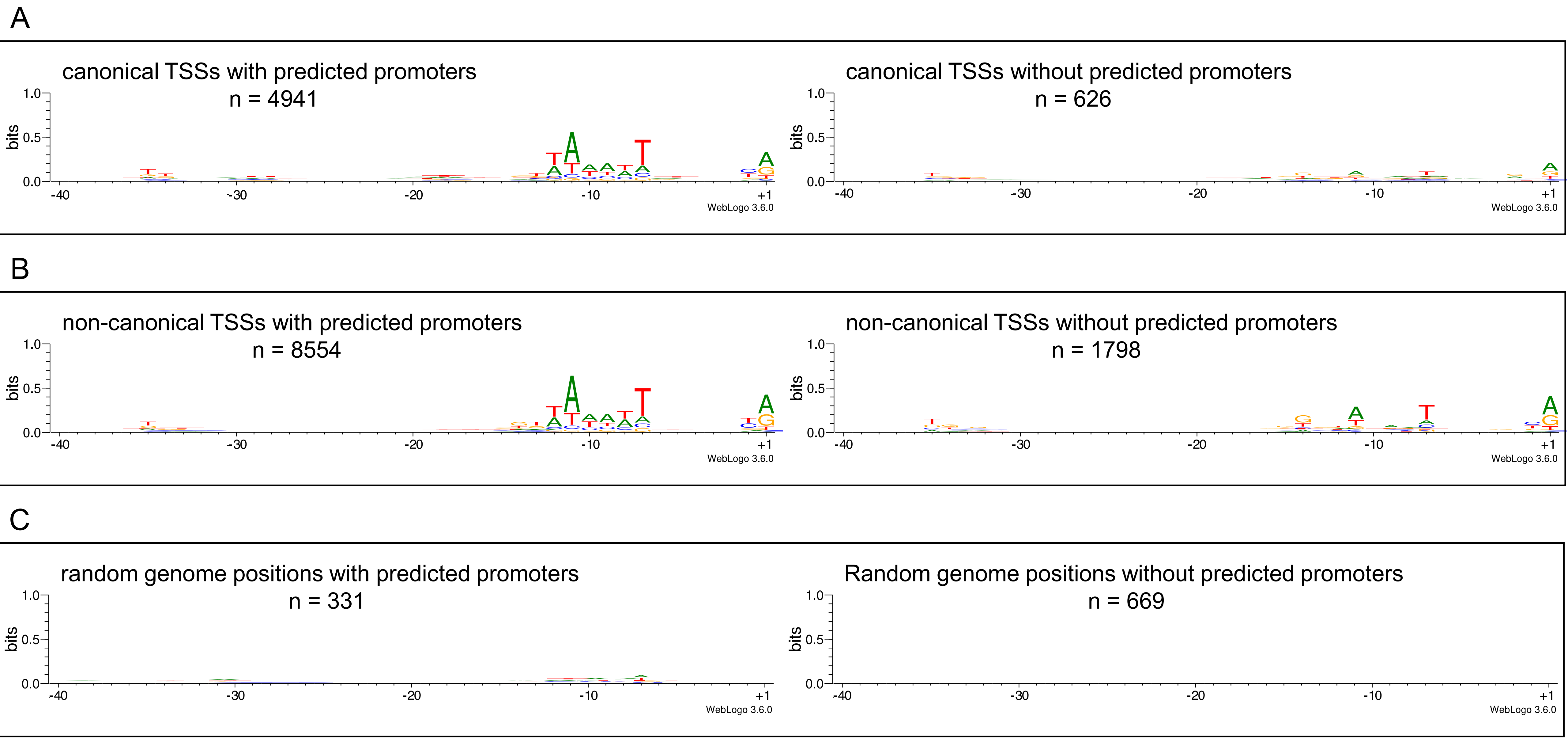

Supplementary Figure S4 Promoter sequence logos for sequences upstream of canonical TSS (**A**), non-canonical TSS (**B**), and random genome positions (**C**). Sequences are sorted into groups with (left panels) and without (right panels) a promoter predicted with the program bTSSfinder. The number of sequences used to create the sequence logo is indicated in each case.

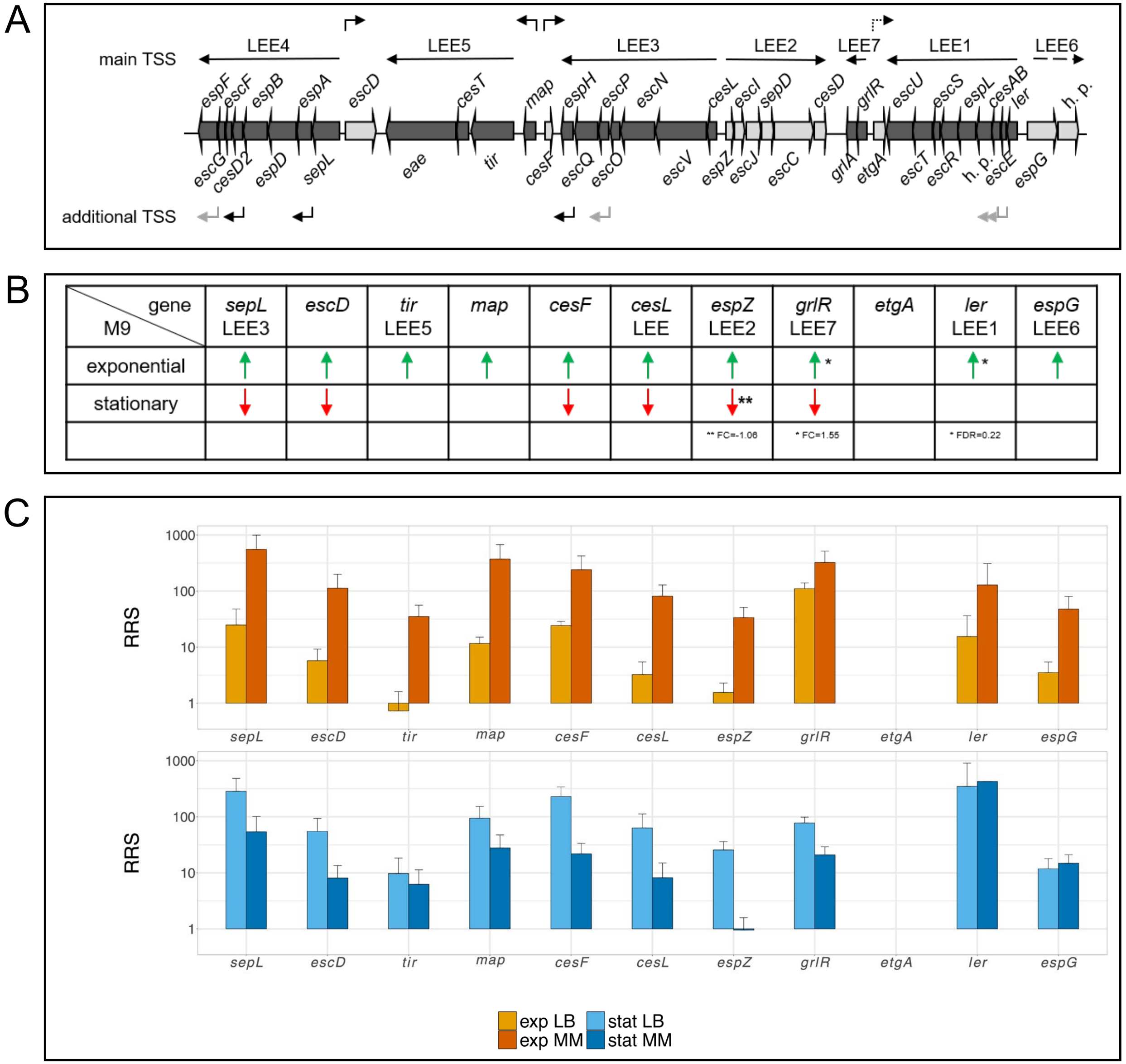

Supplementary Figure S5 Differential expression of the LEE Pathogenicity Island based on Cappable-seq data.

A LEE pathogenicity island of *E. coli* O157:H7 EDL933 (adapted from Gaytán et al., 2016). Gene names are derived from GenBank accession NZ_CP008957, genome annotation 2017/02 (h. p., hypothetical gene). Arrows in the upper part indicate TSS identified with Cappable-seq for polycistronic (LEE1-7, straight arrows) or monocistronic operon structures (*etgA*, *cesF*, *map*, *escD*, angled arrows); dashed arrow, TSS located downstream of assumed start codon; dotted arrow, no reliable TSS. Arrows in the lower part indicate additional TSS identified with Cappable-seq, whereby black arrows represent probably true individual TSS (*espH*, *espA*, *cesD2*) and gray arrows represent weak TSS probably below the expression level of the genomic context (visual inspection for *escE*, *escP, espF*, S/N ratio < 1.5 for *cesAB*).

B Differential expression of TSS for LEE encoded virulence genes. Significant upregulation (green arrows) and downregulation (red arrows) in M9 minimal medium compared to LB medium in exponential or stationary growth phase for TSS upstream of the first gene in the respective operon structure or for the monocistronically expressed genes. Asterisks indicate genes where significance criterium is not met (FDR > 0.05, |FC| < 2, actual values are indicated below). *etgA* showed no reliable TSS and thus was omitted from the analysis.

C Relative read scores of TSS for the main genes of the LEE pathogenicity island. The mean RRS and the positive standard deviation calculated from the replicates are given for exponential (exp, upper panel, orange/red) and stationary (stat, lower panel, light/dark blue) phase in plain LB medium and minimal medium (MM).

Supplementary Figure S6 Volcano plots for transcription start sites of different categories, i.e., gTSS of fAG and hAG, iTSS, asTSS, and oTSS. Differential expression is calculated for a given stress/growth condition compared to non-stress, i.e. altered growth medium in exponential ('exp') or stationary ('stat') growth phase or altered growth phase ('phase'). The $-\log_{10}$ FDR (false discovery rate) is plotted against the $\log_2$ FC (fold change) for the indicated stress situation. Differentially upregulated, downregulated and unchanged (not significantly different) expressed TSS are marked in green, yellow and grey, respectively. Dashed lines and dotted lines indicate limits, i.e., $\log_2\text{FC} > |2|$ and FDR cutoff $> -\log_{10}(0.05)$, respectively.

Stress: Growth medium
Comparison in exponential growth phase

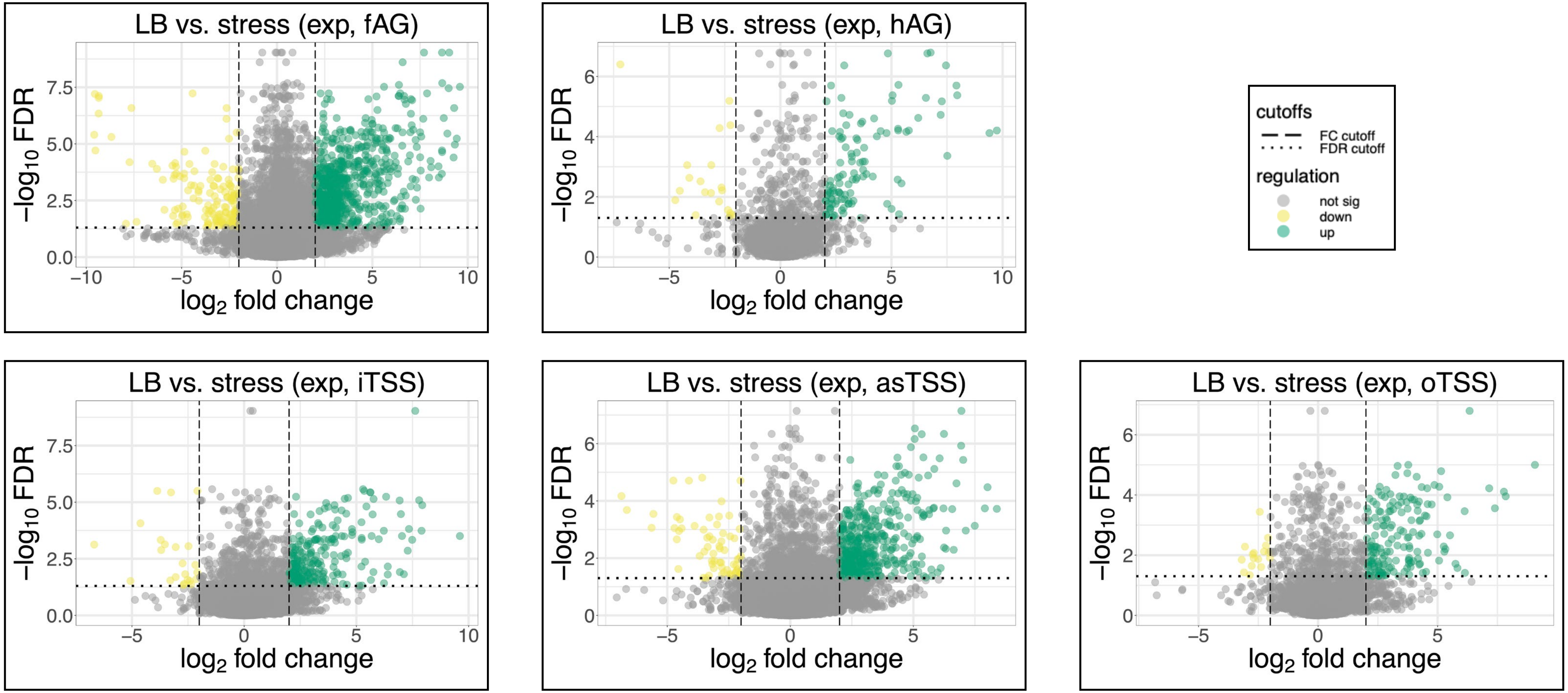

Stress: Growth medium
Comparison in stationary growth phase

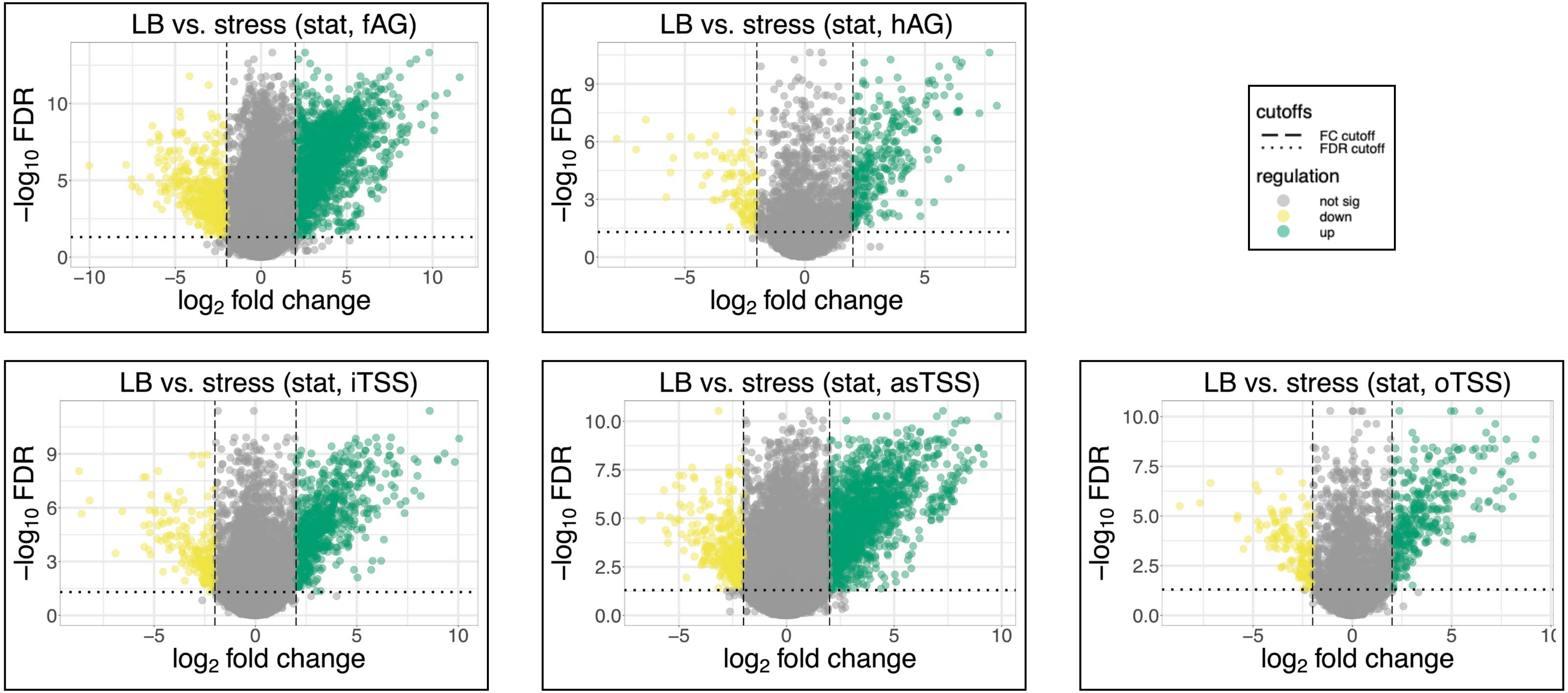

Stress: Growth phase
Comparison exponential vs. stationary

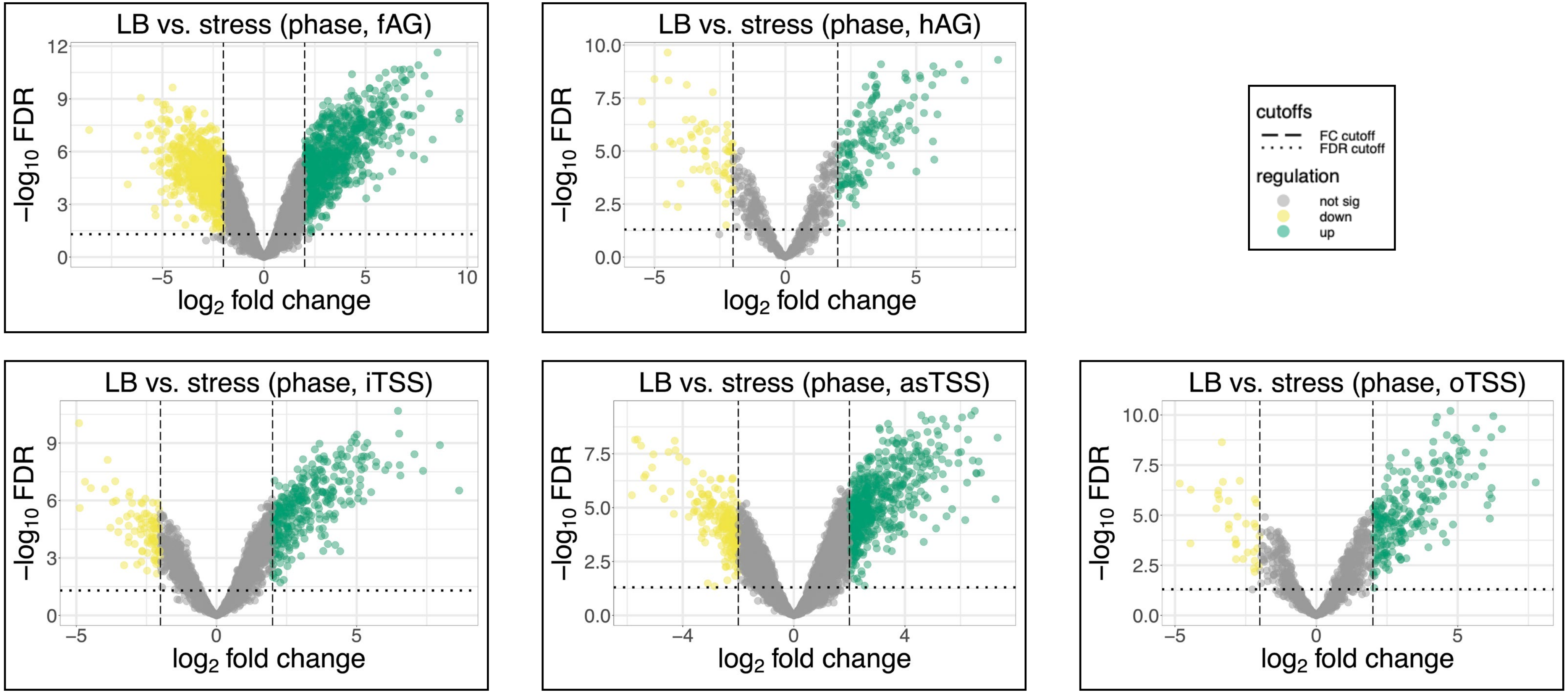

Supplement: Supplementary file 1 — Supplementary Material 1 [file 12866_2023_2988_MOESM1_ESM.pdf]
